# Conserved Responses in a War of Small Molecules between a Plant-Pathogenic Bacterium and Fungi

**DOI:** 10.1128/mBio.00820-18

**Published:** 2018-05-22

**Authors:** Joseph E. Spraker, Philipp Wiemann, Joshua A. Baccile, Nandhitha Venkatesh, Julia Schumacher, Frank C. Schroeder, Laura M. Sanchez, Nancy P. Keller

**Affiliations:** a Department of Plant Pathology, University of Wisconsin—Madison, Madison, Wisconsin, USA; b Department of Medical Microbiology and Immunology, University of Wisconsin—Madison, Madison, Wisconsin, USA; c Boyce Thompson Institute and Department of Chemistry and Chemical Biology, Cornell University, Ithaca, New York, USA; d Institute for Biology and Biotechnology of Plants, Westfälische Wilhelms-Universität Münster, Münster, Germany; e Department of Medicinal Chemistry and Pharmacognosy, College of Pharmacy, University of Illinois at Chicago, Chicago, Illinois, USA; f Department of Bacteriology, University of Wisconsin—Madison, Madison, Wisconsin, USA; Johns Hopkins Bloomberg School of Public Health

**Keywords:** *Fusarium*, antimicrobial activity, bacterial wilt, bikaverin, chemical communication, microbial interactions, ralsolamycin

## Abstract

Small-molecule signaling is one major mode of communication within the polymicrobial consortium of soil and rhizosphere. While microbial secondary metabolite (SM) production and responses of individual species have been studied extensively, little is known about potentially conserved roles of SM signals in multilayered symbiotic or antagonistic relationships. Here, we characterize the SM-mediated interaction between the plant-pathogenic bacterium Ralstonia solanacearum and the two plant-pathogenic fungi Fusarium fujikuroi and Botrytis cinerea. We show that cellular differentiation and SM biosynthesis in F. fujikuroi are induced by the bacterially produced lipopeptide ralsolamycin (synonym ralstonin A). In particular, fungal bikaverin production is induced and preferentially accumulates in fungal survival spores (chlamydospores) only when exposed to supernatants of ralsolamycin-producing strains of R. solanacearum. Although inactivation of bikaverin biosynthesis moderately increases chlamydospore invasion by R. solanacearum, we show that other metabolites such as beauvericin are also induced by ralsolamycin and contribute to suppression of R. solanacearum growth *in vitro*. Based on our findings that bikaverin antagonizes R. solanacearum and that ralsolamycin induces bikaverin biosynthesis in F. fujikuroi, we asked whether other bikaverin-producing fungi show similar responses to ralsolamycin. Examining a strain of B. cinerea that horizontally acquired the bikaverin gene cluster from *Fusarium*, we found that ralsolamycin induced bikaverin biosynthesis in this fungus. Our results suggest that conservation of microbial SM responses across distantly related fungi may arise from horizontal transfer of protective gene clusters that are activated by conserved regulatory cues, e.g., a bacterial lipopeptide, providing consistent fitness advantages in dynamic polymicrobial networks.

## IMPORTANCE

Bacteria and fungi are ubiquitous neighbors in many environments, including the rhizosphere. Many of these organisms are notorious as economically devastating plant pathogens, but little is known about how they communicate chemically with each other. Here, we uncover a conserved antagonistic communication between the widespread bacterial wilt pathogen Ralstonia solanacearum and plant-pathogenic fungi from disparate genera, *Fusarium* and *Botrytis*. Exposure of Fusarium fujikuroi to the bacterial lipopeptide ralsolamycin resulted in production of the antibacterial metabolite bikaverin specifically in fungal tissues invaded by *Ralstonia*. Remarkably, ralsolamycin induction of bikaverin was conserved in a Botrytis cinerea isolate carrying a horizontally transferred bikaverin gene cluster. These results indicate that horizontally transferred gene clusters may carry regulatory prompts that contribute to conserved fitness functions in polymicrobial environments.

## INTRODUCTION

Fungi and bacteria coexist in many ecological settings and are ubiquitous in soil. Although many soil bacteria and fungi are well studied individually because of their importance in agricultural settings, little is known about how they interact with one another. Many soil microbes are equipped with an arsenal of unique biosynthetic enzymes, producing bioactive molecules that are thought to help them secure a niche in their local environment. These compounds are often termed secondary metabolites (SMs) because they are seemingly dispensable in axenic culture, although the genes involved in their production can account for large proportions of some microbial genomes and are evolutionarily maintained, suggesting that they are indispensable in a natural environment. Hence, it is thought that microbial SMs play an important role in intra- and interspecific interactions, including cross-kingdom communication (1–3).

The plant-pathogenic bacterium Ralstonia solanacearum is a common inhabitant of soils globally. It is a devastating pathogen of well over 200 plant species, including both mono- and dicotyledonous plants (4). Although R. solanacearum has been studied primarily because of its severe impacts on plant hosts, it has recently been shown to impact plant-prokaryote community composition (5), and it also interacts intimately with many soil fungi through volatile and diffusible signaling (6–8). The lipopeptide ralsolamycin (synonym ralstonin A), produced by a hybrid polyketide synthase/nonribosomal peptide synthetase (PKS-NRPS) biosynthetic gene cluster (*rmy*) (7, 9, 10), was shown to induce chlamydospore formation in a phylogenetically diverse panel of plant-associated and/or soil-inhabiting fungi representing members of the Mucoromycota, Ascomycota, and Basidiomycota (7). Among the assayed fungi were members of the genus *Fusarium*.

*Fusarium* spp. are well known for their SM biosynthetic capacity, as they produce a number of mycotoxins, such as beauvericin, fumonisins, trichothecenes, zearalenone, and fusaric acid, which impact animal and human health (11). Aside from these well-described toxins, *Fusarium* spp. also produce plant growth-regulatory hormones such as gibberellic acid (12) as well as characteristic colored metabolites, including bikaverin (Fusarium fujikuroi and oxysporum species complex) (13), aurofusarin (Fusarium graminearum species complex) (14), and fusarubins/bostrycoidins (conserved in all characterized *Fusarium* spp. to date) (15–18); some of these SMs have potential applications in medicine (19) and biocontrol (20, 21). While fusarubins/bostrycoidins have been characterized as, perithecial pigments (15–18), the biological and ecological functions of most of these metabolites remain elusive. Surprisingly, a horizontally acquired and functional bikaverin gene cluster is present in some strains of the phylogenetically distinct, broad-range necrotrophic plant pathogen Botrytis cinerea (22).

Here, we describe interactions between R. solanacearum and the fungi F. fujikuroi and B. cinerea, which we show to be mediated by the bacterial lipopeptide ralsolamycin. Ralsolamycin induces visible bikaverin production in several *Fusarium* species, specifically at the bacterial-fungal interaction zone where it accumulates in the chlamydospores. Metabolic profiling of F. fujikuroi-ralsolamycin treatments reveals a unique fungal metabolic shift comprising not only bikaverin but also other compounds, including the bioactive metabolite beauvericin. Both bikaverin and beauvericin suppress R. solanacearum growth, suggesting a protective fungal function of these bacterium-induced SMs against bacterial competition. Remarkably, ralsolamycin retains its function in inducing bikaverin production in B. cinerea despite disparate regulation by nutritional cues of this metabolite in the two fungal species. These findings support a model where regulatory cues responsive to bacterial SM signals can be horizontally transferred in tandem with protective fungal SM gene clusters to confer increased ecological fitness.

## RESULTS

### Bacterial ralsolamycin induces bikaverin production in Fusarium fujikuroi.

**FIG 1:**
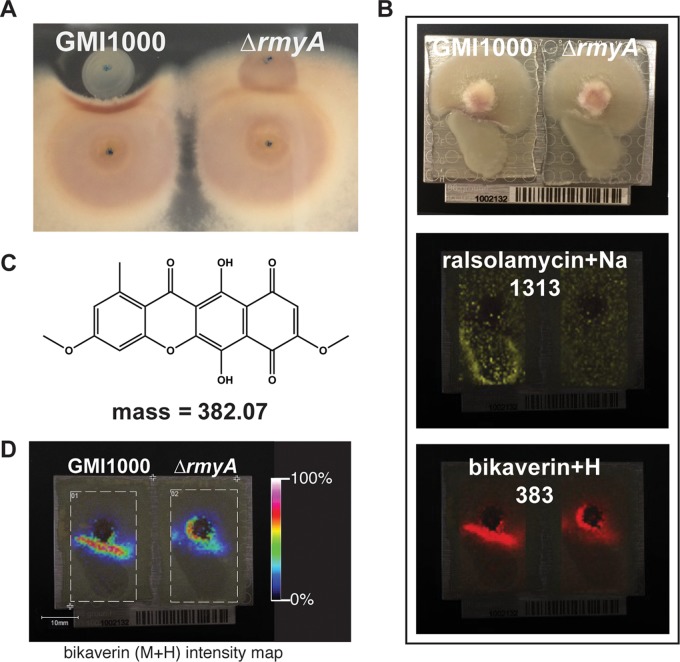
Ralsolamycin induces bikaverin production in F. fujikuroi. (A) When grown in coculture on an agar plate, R. solanacearum GMI1000, but not the Δ*rmyA* mutant, inhibits growth of F. fujikuroi and induces the production of a red pigment in this fungus. (B) Imaging mass spectrometry experiments. (Top) Picture of cocultures mounted on MALDI plate. (Middle) Ralsolamycin (*m/z* = 1,313) is the only detectable difference between GMI1000 and Δ*rmyA* strains. (Bottom) Imaging mass spectrometry shows that bikaverin (*m/z* = 383) is produced in proximity to bacterial colonies. The complete IMS data set is shown in Fig. S2. (C) Chemical structure of bikaverin and exact mass. (D) Intensity map of bikaverin shows that substantially more compound accumulates proximal to the GMI1000 colony than to the Δ*rmyA* colony.

The ralsolamycin-producing wild-type R. solanacearum strain GMI1000 can induce chlamydospore development in many fungi, including F. fujikuroi, F. graminearum, Fusarium verticillioides, and Fusarium oxysporum f. sp. lycopersici (7). Using an *in vitro* coculture assay (Fig. 1A), we found that ralsolamycin (see Fig. A1 and Table A1 in Data Set S1 in the supplemental material) also induces pigment production in three *Fusarium* species: F. fujikuroi, F. graminearum, and F. oxysporum f. sp. lycopersici (Fig. S1A). Conversely, the Δ*rmyA* mutant did not induce chlamydospore development or localized pigment production in the coincubated fungal species (Fig. S1A).

10.1128/mBio.00820-18.2 FIG S1  Ralsolamycin induces bikaverin production in F. fujikuroi. (A) Cocultures of GMI1000 induce pigment production in F. graminearum, F. oxysporum f. sp. lycopersici, F. fujikuroi, and B. cinerea, while the Δ*rmyA* mutant defective in ralsolamycin production shows no such effect. (B) Expanded IMS data set showing masses that could clearly be identified as associated with microbial culture. All numbers in white represent the *m/z* (±0.5) for that picture. The picture labeled 200 to 2,500 represents the signals from the entire data set, showing areas in black where no data were collected. The last picture on the right shows the original image upon which the masses were mapped. In all pictures, GMI1000 is on the left and the Δ*rmyA* strain is on the right. *m/z* 1,313 shows localization of ralsolamycin (M+Na^+^). *m/z* 307, 328, and 345 are included to show the absence of signal for fusarubin at the interaction zone. Download FIG S1, TIF file, 7.4 MB. Copyright © 2018 Spraker et al. 2018 Spraker et al. This content is distributed under the terms of the Creative Commons Attribution 4.0 International license.

10.1128/mBio.00820-18.10 DATA SET S1  An appendix showing spectroscopy data. Download DATA SET S1, PDF file, 0.2 MB. Copyright © 2018 Spraker et al. 2018 Spraker et al. This content is distributed under the terms of the Creative Commons Attribution 4.0 International license.

10.1128/mBio.00820-18.3 FIG S2  Bikaverin accumulates in F. fujikuroi chlamydospores. Under low-nitrogen conditions, most F. fujikuroi chlamydospores are highly pigmented with bikaverin, while under high-nitrogen conditions, chlamydospores are largely hyaline, but some contain faint amounts of pigmentation. White arrowheads indicate chlamydospores with bikaverin. Red arrowheads indicate bikaverin accumulation in hyphae. Download FIG S2, TIF file, 3.3 MB. Copyright © 2018 Spraker et al. 2018 Spraker et al. This content is distributed under the terms of the Creative Commons Attribution 4.0 International license.

We selected F. fujikuroi strain IMI58289 for more detailed investigations because of its well-annotated genome and SM biosynthetic potential (17). Additionally, the biosynthetic pathways and molecular cues that regulate production of the two red pigments produced by this isolate, bikaverin (13) and fusarubin (15), are fairly well understood. The interacting R. solanacearum/F. fujikuroi colonies were excised at 72 h when pigmentation was observed at the zone of interaction and analyzed via matrix-assisted laser desorption ionization–time of flight imaging mass spectrometry (MALDI-TOF IMS) (23) to map the spatial distribution of metabolites to their specific location in the coculture. In total, 30 ions mapped to distinct locations within microbial cultures (Fig. S1B). As expected, R. solanacearum GMI1000 showed ions representing the sodium adduct of ralsolamycin, which were absent in *ΔrmyA* cultures (7) (Fig. 1B). Nine ions mapped specifically to the area of pigment production at the GMI1000-F. fujikuroi interface (Fig. S1B). Three of these ions, *m/z* 383, 405, and 421, were different adducts (proton, sodium, and potassium adducts, respectively) of one compound with a molecular mass of 382 Da (±0.5 Da), corresponding to that of the polyketide bikaverin (13) (Fig. 1B and C and S1B). These signals were more intense at the region where F. fujikuroi was interacting with GMI1000 than where it interacted with the Δ*rmyA* mutant, which mainly showed an intensified signal at the center of the fungal colony (Fig. 1D). Two other ions, *m/z* 369 and 385, were putatively identified as sodium and potassium adducts, respectively, of gibberellic acid 3 (GA3, molecular weight [MW] of 346 [Fig. S1B]). These ions were diffused across the colony and were detected in the interaction of F. fujikuroi with both R. solanacearum GMI1000 and the Δ*rmyA* mutant. Four other ions detected in the interaction zone could not be assigned to any known F. fujikuroi metabolites. We did not observe any specific signals for fusarubin production (MW of 306 [Fig. S1B]), suggesting that the red pigmentation at the coculture interface was primarily due to bikaverin production.

### Bikaverin accumulates in chlamydospores and contributes to protection from invasion.

**FIG 2:**
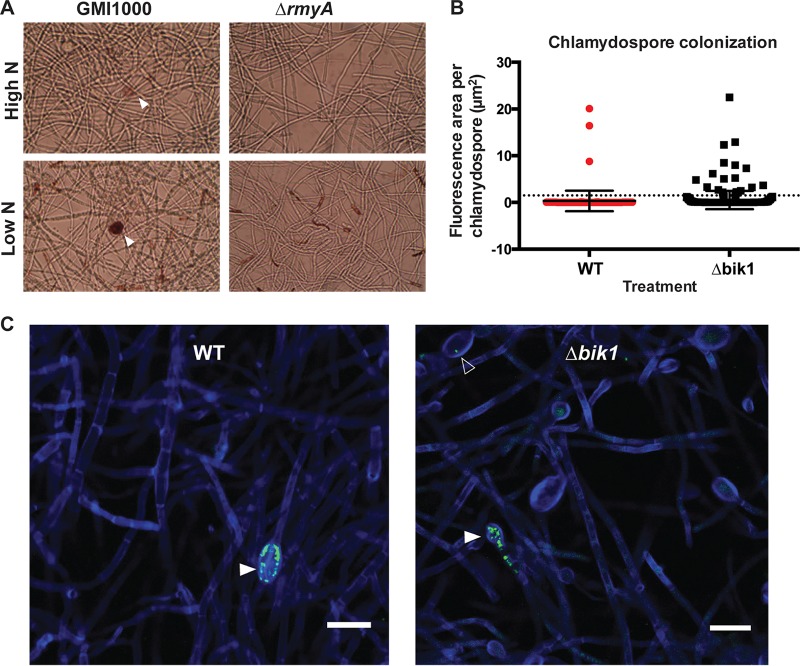
Bikaverin contributes to protecting chlamydospores from bacterial invasion. (A) Microscopic examination of chlamydospores shows bikaverin accumulation in chlamydospores induced by GMI1000-conditioned medium under both high- and low-nitrogen conditions. (B) Analysis of confocal microscopy images for total bacterial GFP fluorescence in all fungal chlamydospores shows no significant change in total bacterial burden (*P* = 0.315) between Δ*bik1* (*n* = 301) and wild-type (WT) (*n* = 154) F. fujikuroi. The dotted line indicates the 1.5-µm^2^-area cutoff used for chi-square test of colonization differences, showing that more Δ*bik1* chlamydospores are invaded, although at low bacterial counts. (C) Confocal microscopy of GFP-labeled bacteria in chlamydospores of wild-type (WT) and Δ*bik1* isolates of F. fujikuroi. Solid white arrowheads indicate heavily colonized chlamydospores; an empty white arrowhead indicates invasion by single bacterial cells. Fungal cell walls (blue) are stained with calcofluor white, and GMI1000 cells are constitutively expressing GFP (green). Bars, 20 µm. All Z-sections are set to 0.5 µm.

Focusing on bikaverin due to its clear response to ralsolamycin, we aimed to identify the cellular location of bikaverin accumulation in *Fusarium*, particularly regarding chlamydospore formation. Chlamydospores are long-lived survival spores that we previously found to be induced in many fungi by ralsolamycin (7). Microscopic examination of GMI1000-F. fujikuroi cocultures revealed red-pigmented chlamydospores (Fig. 2A and S2), indicating that bikaverin accumulated specifically in these fungal survival structures. This contrasted with a near-loss of chlamydospore production and no pigmentation in the Δ*rmyA* mutant-F. fujikuroi interaction (Fig. 2A).

Previous microscopic analyses of Aspergillus flavus chlamydospores showed that the spores can be invaded by R. solanacearum (7). Since bikaverin accumulates at high concentrations in the chlamydospores of F. fujikuroi, we hypothesized that this may serve a protective function for chlamydospores against bacterial invasion. Therefore, we set out to compare bacterial colonization of chlamydospores using a green fluorescent protein (GFP)-labeled strain of GMI1000 in coculture with the F. fujikuroi wild type and a *bik1* mutant deficient in bikaverin production (13), using confocal laser scanning microscopy (CLSM). We found GFP-labeled bacteria in 1.95% of wild-type and 6.65% of Δ*bik1* chlamydospores. A chi-square analysis of colonized (containing one or more bacteria) versus uncolonized (containing no bacteria) chlamydospores of the wild-type and Δ*bik1* strains showed colonization to be dependent on bikaverin production (χ^2^ = 4.682, *K* = 1, *P* = 0.031 [Table S1]). Although this analysis suggests that bikaverin contributes to protecting chlamydospores from invasion, the mean total fluorescence areas inside all of the observed chlamydospores were not significantly different between the wild type and the Δ*bik1* strain (*p* = 0.315) (Fig. 2B). The wild-type F. fujikuroi strain had a few heavily colonized chlamydospores similar to those of the Δ*bik1* strain but had fewer colonized with low bacterial numbers (Fig. 2B and C). In total, these data indicate that while bikaverin production can reduce bacterial invasion, it is not solely responsible for limiting bacterial growth in invaded F. fujikuroi chlamydospores and that other fungal metabolites likely contribute to protection against bacterial invasion and/or proliferation.

10.1128/mBio.00820-18.6 TABLE S1  Chi-square contingency table. “Bac+” indicates colonized chlamydospores, while “Bac−” indicates uncolonized chlamydospores. Download TABLE S1, PDF file, 0.04 MB. Copyright © 2018 Spraker et al. 2018 Spraker et al. This content is distributed under the terms of the Creative Commons Attribution 4.0 International license.

### Ralsolamycin overrides suppression of multiple SMs under high-nitrogen conditions.

**FIG 3:**
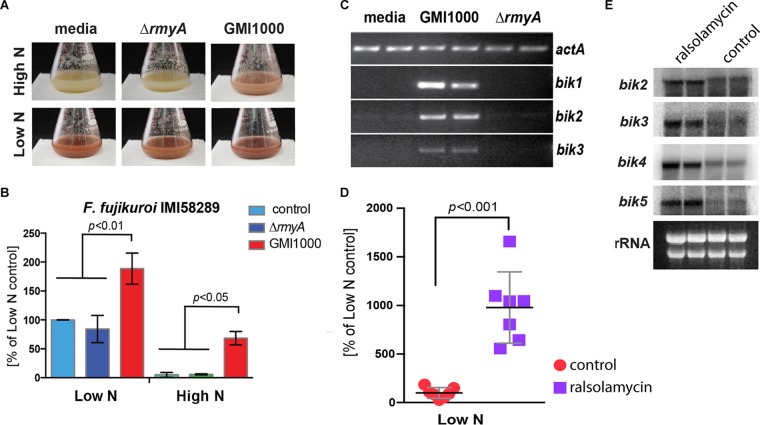
GMI1000-conditioned medium and purified ralsolamycin induce bikaverin production in F. fujikuroi. (A) GMI1000-conditioned medium, but not Δ*rmyA* strain-conditioned medium, caused dramatic shifts in pigment production in both F. fujikuroi strains relative to medium controls. (B) HPLC-PDA analysis of liquid cultures of F. fujikuroi treated with GMI1000-conditioned medium shows significantly increased bikaverin production under both high- and low-nitrogen conditions. (C) Semiquantitative reverse transcription-PCR shows that bikaverin biosynthetic genes *bik1*, *bik2*, and *bik3* in F. fujikuroi are induced in response to GMI1000-conditioned medium but not in response to Δ*rmyA* strain-conditioned medium or bacterial medium controls. Actin was used as a loading control. (D) Purified ralsolamycin induces bikaverin production in F. fujikuroi which was quantified by HPLC from seven biological replicates. Acetonitrile was used as the carrier control. (E) Northern analysis showing induction of bikaverin biosynthetic genes (technical duplicates) in response to purified ralsolamycin.

Although the ecological functions of most fungal SMs have not been established, the production of many SMs, including bikaverin, in F. fujikuroi is tightly regulated by multiple abiotic signals, including pH and nitrogen availability (13, 17). To determine if ralsolamycin can override the tight nitrogen-dependent regulation of the bikaverin biosynthetic gene cluster where bikaverin is repressed by high nitrogen, we grew F. fujikuroi under either high- or low-nitrogen conditions, adding R. solanacearum-conditioned medium from either GMI1000 or the Δ*rmyA* mutant. Additionally, we supplemented F. fujikuroi cultures with purified ralsolamycin to test bikaverin induction. High-performance liquid chromatography (HPLC) and expression analyses showed that, regardless of nitrogen regime, GMI1000 extracts (Fig. 3A to C) and purified ralsolamycin (Fig. 3D and E) significantly increased bikaverin production through transcriptional activation of the *bik* cluster genes. In fact, bikaverin levels in the high-nitrogen/GMI1000 treatment approached that of the wild type in low nitrogen. Taken together, this suggests that ralsolamycin is a primary cue in inducing *bik* gene expression and bikaverin biosynthesis, regardless of nutritional environment.

To further explore the fungal chemical repertoire induced by ralsolamycin, we analyzed fungal cultures using ultrahigh-performance liquid chromatography coupled to high-resolution mass spectrometry (UHPLC-HRMS) using the program XCMS (49). Principal-component analyses of all samples showed that F. fujikuroi metabolism is shifted most in response to GMI1000-conditioned, high-nitrogen medium (Fig. S3A). Using the metaXCMS package (24), we found that, in accordance with previous studies (17), a low-nitrogen condition induces far more spectral features (573) (Fig. S3B) than it represses (207) (Fig. S3C) compared to high-nitrogen conditions. However, treatment with GMI1000-conditioned high-nitrogen medium induced a total of 311 features compared to unconditioned high-nitrogen medium alone (Fig. S3B), restoring, as shown in Fig. 3, bikaverin. When Δ*rmyA* mutant-conditioned high-nitrogen medium was assessed, only 114 features were found to be induced compared to unconditioned high-nitrogen medium (Fig. S3B), indicating that this treatment caused the least drastic shift in metabolism. Interestingly, we found the highest number of common induced features between unconditioned low-nitrogen medium and GMI1000-conditioned high-nitrogen medium compared to unconditioned high-nitrogen medium (25) (Fig. S3B).

10.1128/mBio.00820-18.4 FIG S3  Ralsolamycin overrides suppression of multiple SMs under high-nitrogen conditions. (A) Score plot of principal-component analyses (PCA) of all samples in our LC-MS data set shows that nitrogen availability explains the largest shift in metabolism to F. fujikuroi. Among the high-nitrogen samples, GMI1000 treatment induces a substantial shift in the metabolic profile. Colored ovals represent the spread of the data points. Data represent extracts from four biological replicates. (B) Venn diagram showing overlap in upregulated metabolites of each condition relative to high-nitrogen medium control. Bikaverin is among the 42 metabolites similarly increased both in GMI1000 and under low-nitrogen conditions. The red circle highlights differential metabolites from high nitrogen (high-N) versus low nitrogen (low-N). (C) Venn diagram showing overlap in downregulated metabolites of each condition relative to high-nitrogen medium control. (D) Bikaverin and beauvericin are significantly induced in response to ralsolamycin produced by GMI1000 under otherwise repressive conditions (high nitrogen). Graphs represent area top intensity as calculated in MAVEN, from four biological replicates. * and ** indicate adjusted *P* values of <0.05 and <0.01, respectively, from ANOVA and Tukey’s multiple-comparison tests. Download FIG S3, TIF file, 1.6 MB. Copyright © 2018 Spraker et al. 2018 Spraker et al. This content is distributed under the terms of the Creative Commons Attribution 4.0 International license.

Targeted metabolomics examination of these treatments revealed a significant increase in GMI1000 treatment of not only bikaverin (*P* = 0.003) but also the cytotoxic metabolite beauvericin (*P* = 0.008) (Table S2 and Fig. S3). Like bikaverin, the biosynthetic genes of beauvericin are known to be repressed under high-nitrogen conditions, although via a different regulatory mechanism than bikaverin (13, 17, 26).

10.1128/mBio.00820-18.7 TABLE S2  Targeted metabolomics assessment of changes in F. fujikuroi SM production in response to ralsolamycin. Changes were determined by comparing relative M+H peak area tops in the program MAVEN, from four biological replicates. Download TABLE S2, PDF file, 0.1 MB. Copyright © 2018 Spraker et al. 2018 Spraker et al. This content is distributed under the terms of the Creative Commons Attribution 4.0 International license.

### Antibacterial activity of bikaverin and beauvericin.

The induction of both bikaverin and beauvericin in response to the bacterial signal ralsolamycin, coupled with the accruement of bikaverin in chlamydospores, suggested a possible evolved function of these metabolites toward protection from the endosymbiotic bacteria. Indeed, both metabolites have been previously shown to be antibacterial (27–30).

**FIG 4:**
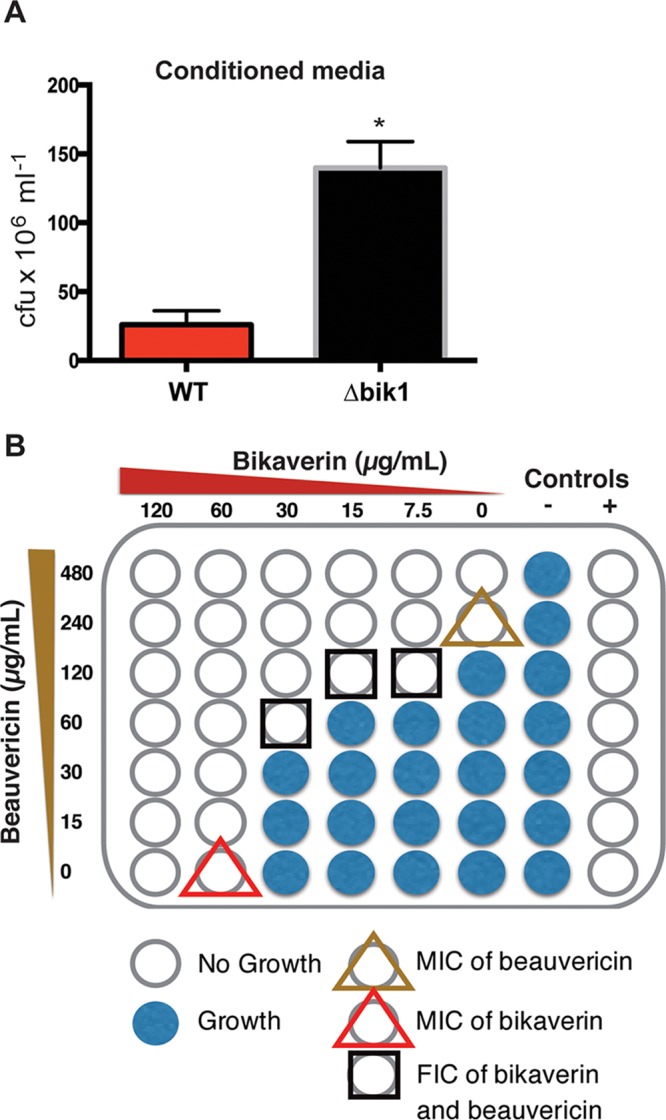
*Fusarium* SMs contribute to protecting chlamydospores from bacterial invasion. (A) F. fujikuroi (wild-type [WT])-conditioned medium significantly reduced recoverable colonies of R. solanacearum relative to conditioned medium from a Δ*bik1* mutant. (B) Diagram showing the combinations of concentrations of bikaverin and beauvericin used in the checkerboard assay and the observed MICs and FICs of these compounds toward R. solanacearum.

To examine this hypothesis, we first used conditioned medium obtained from F. fujikuroi wild type and the *bik1* mutant for antibacterial activity assays and found that wild-type F. fujikuroi-conditioned medium reduced cell viability by 5-fold relative to the Δ*bik1* mutant-conditioned medium (Fig. 4A). We then tested the antibacterial activity and interactions between bikaverin and beauvericin against R. solanacearum. This was performed with a checkerboard analysis (31), which showed the MICs of bikaverin and beauvericin to be 60 (157 µM) and 240 (306 µM) µg/ml, respectively. The bikaverin MIC was higher than that reported for Escherichia coli of 65 µM (27), and the beauvericin MIC was higher than the reported 32 µM for Lactobacillus acidophilus and Staphylococcus aureus (28). The interaction between the metabolites was characterized as additive due to its fractional inhibitory concentration (FIC) index value of 0.708 (Fig. 4B; Table S3). These data, in combination with our metabolomics data, support the hypothesis that bikaverin and other SMs, such as beauvericin, act together to protect F. fujikuroi from bacterial invasion and/or proliferation.

10.1128/mBio.00820-18.8 TABLE S3  Checkerboard results showing additive interaction between bikaverin and beauvericin against Ralstonia solanacearum GMI1000. Download TABLE S3, PDF file, 0.3 MB. Copyright © 2018 Spraker et al. 2018 Spraker et al. This content is distributed under the terms of the Creative Commons Attribution 4.0 International license.

### Conservation of ralsolamycin-dependent induction of the horizontally acquired bikaverin cluster in Botrytis cinerea.

**FIG 5:**
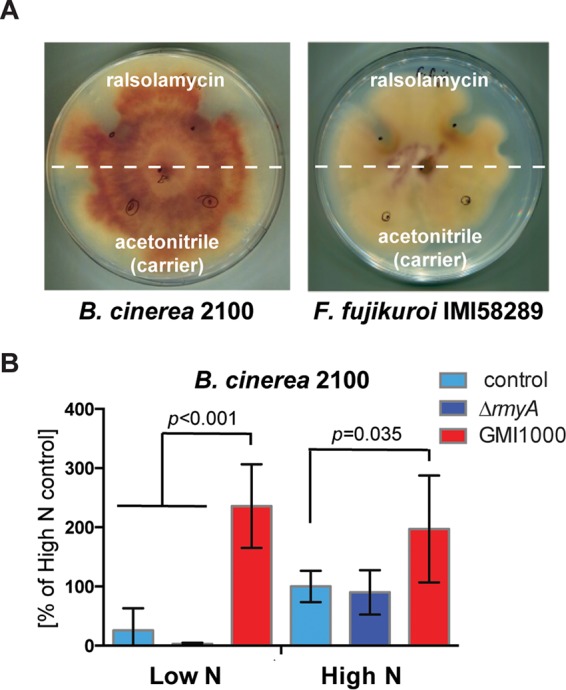
Ralsolamycin induces bikaverin production in both F. fujikuroi and B. cinerea. (A) Purified ralsolamycin induces bikaverin production in both F. fujikuroi and B. cinerea. The white dashed line separates treatments and controls. Solutions were applied at black dots shown on plate. (B) HPLC-PDA analysis of liquid cultures of B. cinerea treated with GMI1000-conditioned medium shows increased bikaverin production under both high- and low-nitrogen conditions. Pictures of culture flasks can be seen in Fig. S4C.

We hypothesized that if ralsolamycin serves as an “alert” signal of invading endosymbiotic bacteria, then it may also induce the protective SM bikaverin in other fungi. Most bikaverin-producing fungi belong to the genus *Fusarium* (class Sordariomycetes), so we first confirmed that ralsolamycin induced bikaverin production in the congeneric plant pathogen F. oxysporum f. sp. lycopersici (Fig. S4A). Interestingly, some isolates of the taxonomically distant fungus B. cinerea (class Leotiomycetes) have also acquired and maintained the bikaverin gene cluster through an ancient horizontal transfer event from *Fusarium* (22, 32). We therefore set out to determine if bikaverin synthesis was affected by ralsolamycin in the B. cinerea strain 2100, which contains a functional bikaverin gene cluster (22). First, we tested whether purified ralsolamycin has similar effects on bikaverin production in the two fungal species. When grown on solid medium, purified ralsolamycin caused significant growth inhibition of F. fujikuroi and B. cinerea with accumulating red pigment in the zone of interaction compared to the control (Fig. 5A). Although B. cinerea is a species that produces few to no chlamydospores, microscopic examination of the B. cinerea culture challenged with GMI1000 showed ralsolamycin-dependent accumulation of bikaverin in intra- and extrahyphal agglomerates as well as a few chlamydospores (Fig. S4B).

10.1128/mBio.00820-18.5 FIG S4  Ralsolamycin induces bikaverin production in Fusarium oxysporum and Botrytis cinerea. (A) Under low-nitrogen conditions, GMI1000-conditioned medium induces a nearly 4-fold increase in bikaverin production in F. oxysporum f. sp. lycopersici. (B) Under all conditions, B. cinerea chlamydospores were only occasionally observed. Under high-nitrogen conditions, bikaverin accumulated in hyphal agglomerates, while under low-nitrogen conditions, hyphae are generally hyaline, except for in GMI1000-conditioned medium, where bikaverin accumulated in hyphal agglomerates and occasional chlamydospores. The white arrowheads indicate points of bikaverin accumulation. (C) Representative photos of culture flasks of B. cinerea showing increased accumulation of bikaverin under both high- and low-nitrogen conditions when grown in GMI100-conditioned medium. Download FIG S4, TIF file, 4.6 MB. Copyright © 2018 Spraker et al. 2018 Spraker et al. This content is distributed under the terms of the Creative Commons Attribution 4.0 International license.

We next examined the effects of different nutritional conditions on bikaverin production in B. cinerea as we did for F. fujikuroi, using two nitrogen supplementation regimes, both treated with conditioned medium either from GMI1000 or Δ*rmyA* cultures. Analogous to the response of F. fujikuroi, GMI1000-conditioned B. cinerea cultures significantly increased bikaverin production, independent of nitrogen availability, whereas Δ*rmyA* mutant-conditioned medium had no effect (Fig. 5B and S4C). In contrast, changes in the availability of nitrogen had the opposite effect on bikaverin production in B. cinerea compared to F. fujikuroi (Fig. 3B and 5B). Thus, the response to ralsolamycin and its ability to override endogenous nitrogen SM regulatory systems are conserved in two distantly related fungal genera sharing the horizontally transferred bikaverin gene cluster. These data suggest that intermicrobial signals may be an ancestral factor in the regulation of SM clusters that, furthermore, can be transferred along with the SM cluster itself in horizontal transfer events.

## DISCUSSION

Several recent studies of bacterial-fungal interactions have demonstrated shifts in SM production in response to coculture of bacteria and fungi (2, 3, 33–35). These shifts in metabolism indicate that SMs have profound impacts on the ability of these microbes to colonize and survive in complex biotic environments and that microbes respond to specific SM signals as part of a complex network of interactions. Although previous studies have described cross-kingdom induction of SMs, e.g., in Aspergillus nidulans/Streptomyces hygroscopicus (34) or Fusarium tricinctum/Bacillus subtilis (36) pairings, knowledge of mechanisms and molecular players underlying these interactions remains scarce. We recently described a unique *Ralstonia-*Aspergillus flavus interaction where ralsolamycin suppressed synthesis of an A. flavus germination and antibacterial peptide, imizoquin, suggestive of a bacterial advantage in this encounter (8). In contrast, our present study implicates ralsolamycin in the specific induction of antibacterial fungal SMs, including the known metabolites bikaverin and beauvericin, in F. fujikuroi and Botrytis cinerea. Bikaverin is specifically localized to chlamydospores in F. fujikuroi, where it may contribute to protection from bacterial invasion, and is localized to hyphal tissues in B. cinerea, a species that forms few chlamydospores. Significantly, the horizontally transferred bikaverin gene cluster in B. cinerea positively responds to ralsolamycin similarly to the bikaverin cluster in F. fujikuroi, despite having an inverse response to nitrogen sufficiency.

**FIG 6:**
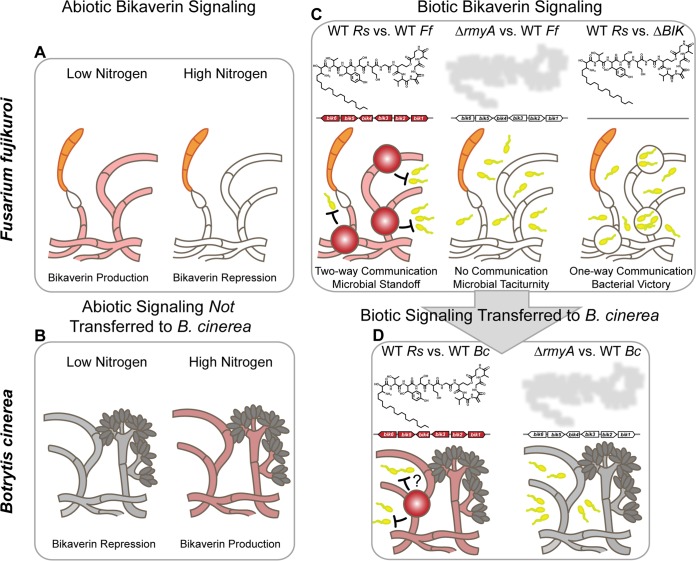
Model of horizontally transferred microbial weaponry. (A) In F. fujikuroi IMI58289, bikaverin biosynthesis is induced under nitrogen-limiting conditions. (B) This abiotic signaling effect on bikaverin production is not conserved in B. cinerea 2100, where the bikaverin production is repressed under nitrogen-limiting conditions. (C) F. fujikuroi IMI58289 induces chlamydospore formation and bikaverin production in response to ralsolamycin produced by R. solanacearum GMI1000 in contrast to the nonproduction mutant (Δ*rmyA*). R. solanacearum GMI1000 is better able to survive in the presence of a bikaverin deletion mutant (Δ*BIK*) than the wild type. (D) In contrast to the nonconserved response to nitrogen availability, the response to ralsolamycin is conserved in B. cinerea 2100. *Rs*, R. solanacearum (yellow cells); *Ff*, *Fusarium fujikuroi* (orange spores); *Bc*, B. cinerea (gray spores).

Since the initial realization that fungi, like bacteria, usually organize the genetic machinery for bioactive SMs in biosynthetic gene clusters (37), considerable efforts have been spent in trying to understand the origins and functions of these units. A relatively recent focus has been on horizontal transfer events where several clusters have been transferred in their (near) entirety across phylogenetically diverse taxa (22, 32, 38), including the bikaverin cluster from *Fusarium* (class Sordariomycetes) to the evolutionarily distant *Botrytis* (class Leotiomycetes) (32). The high level of conservation in terms of gene order and orientation in both lineages, as well as the lack of a *bik* cluster homologue in Sclerotinia sclerotiorum, a close relative of B. cinerea, allowed for a prediction of when the transfer occurred, namely, after evolutionary divergence of *Sclerotinia* and *Botrytis* (32). However, why the bikaverin cluster (or any SM cluster) would be transferred and—if transferred—whether environmental regulatory signals are maintained remained unknown. Our study provides original insights in the evolutionary rationale underlying these events by demonstrating (i) a protective role of the ralsolamycin-induced bikaverin in defending *Fusarium* from bacterial ingress and (ii) conservation of bikaverin induction by ralsolamycin in a distantly related fungus that anciently acquired the *bik* cluster via horizontal gene transfer. We propose that conserved regulation of protective, horizontally transferred SM clusters can provide fitness advantages to fungi. Although our work exemplifies a bacterial/fungal sparring match using chemicals as foils (Fig. 6), we posit that other horizontally transferred SM regulatory responses may be similarly maintained when advantageous to the recipient organism, such as melanin cluster induction by UV stress.

The conservation of the ralsolamycin response in both species extended to overriding disparate bikaverin responses to nitrogen availability in the two fungal genera. Nitrogen availability has been shown to impact the regulation of many F. fujikuroi SM clusters (17, 26), including bikaverin. For example, bikaverin (13), beauvericin (26), and gibberellins (39) are produced only under low-nitrogen conditions but repressed under high-nitrogen conditions (17, 40). The experiments conducted here demonstrate that nitrogen repression of both bikaverin and beauvericin can be overridden by ralsolamycin. Likewise, ralsolamycin overrides repressive bikaverin conditions in B. cinerea (which does not contain the beauvericin gene cluster); however, in this case the repressive conditions are nitrogen limitation, opposite to the conditions that repress bikaverin in *Fusarium*. Taken together, these results suggest that an entire response pathway (e.g., defensive SM production induced by a bacterial SM signal) can be transmitted along with, or in consequence of, a horizontal gene cluster transfer event. In addition to bikaverin and beauvericin, we found that ralsolamycin induced a multitude of unknown F. fujikuroi metabolites under repressive conditions. These data suggest that the ralsolamycin defense signaling pathway can activate other SM clusters which may work additively or synergistically with the antibacterial activity of bikaverin and beauvericin.

This study illustrates microbial small-molecule communications, subsequent microbial responses, and conservation of response in a horizontal transfer event, thereby presenting an archetype of a coupled bacterial-fungal chemically driven feedback network. This work extends our understanding of the intricacies of microbial chemical exchange and how it may shape microbial community dynamics. Although our focus was solely on bacterial-fungal interactions in this work, we consider it likely that such chemical warfare also impacts interactions with plant hosts, potentially driving important plant phenotypes such as nutrient acquisition, growth promotion, and disease outcomes. Ongoing work is aimed at understanding these complex, multipartite interactions and how they may be leveraged to improve plant health.

## MATERIALS AND METHODS

### Strains.

For bacterial strains, R. solanacearum strain GMI1000, the Δ*rmyA* mutant, and the green fluorescent protein-labeled strain were described previously (7). For fungal strains, F. fujikuroi wild-type strain IMI58289 (17) and the Δ*bik1* (previously Δ*pks4*) mutant were described previously (41). F. oxysporum f. sp. lycopersici strain 4287 has been described previously (42). F. graminearum PH1 was obtained from the Fungal Genetic Stock Center. Botrytis cinerea strain 2100 (CECT2100; Spanish Type Culture Collection, Universidad de Valencia) was described previously (43).

### Medium and growth conditions and coculture experiments.

Ralstonia solanacearum strains were routinely grown at 30°C on Casamino Acids-peptone-glucose (CPG) agar (44) supplemented with 1 g/liter yeast extract and on tetrazolium chloride (TZC) (Chem-Impex). Red, mucoid colonies were selected for use in experiments. Liquid bacterial cultures were grown overnight in CPG at 30°C and 250 rpm. Overnight liquid cultures of R. solanacearum were pelleted by centrifugation; washed in equal volumes of sterilized, double-distilled water; quantified using values of optical density at 600 nm (OD_600_); and adjusted to ~2 × 10^8^ cells ml^−1^. For solid-plate experiments, 5 µl (1 × 10^6^ cells) of bacterial suspension was spotted at each point. For R. solanacearum-conditioned medium (both GMI100 and Δ*rmyA* strain) experiments, 1 ml of overnight culture was inoculated into a 500-ml flask containing 250 ml CPG broth and was grown for 24 h at 30°C at 250 rpm. Cells were pelleted by centrifugation in a Sorvall RC 6 Plus centrifuge at 10,000 rpm. Supernatants were sterilized by vacuum filtration through a Nalgene Rapid-Flow 0.2-µm filter unit (Thermo Fisher, Rochester, NY) before being added to F. fujikuroi cultures.

*Fusarium* spp. were routinely grown at 30°C on potato dextrose agar (PDA), and three ~0.5-cm agar plugs were used as inoculum for liquid cultures. For all cultures, F. fujikuroi was grown for 72 h at 28°C in 300-ml flasks containing 100 ml Darken medium (45) and shaken at 180 rpm. Darken starter cultures (DSC) were used as initial inoculum onto plates as well as into liquid medium for bikaverin analysis. For plate assays and MALDI-TOF IMS experiments, 20 µl of DSC was spotted onto the International Streptomyces Project (ISP2) agar 2 cm away from the R. solanacearum spot on CPG plates using wide-end pipette tips. The droplets were dried in a Nuaire biological safety cabinet (NU425-400) to prevent running, and then plates were wrapped with Parafilm once and incubated at room temperature for 72 h prior to imaging and MALDI-TOF IMS experimentation. When assaying purified ralsolamycin, fungi were handled as previously described and either a solution of ralsolamycin in acetonitrile (ACN) or acetonitrile carrier control was applied next to the growing culture of fungi. Droplets were allowed to dry in a laminar flow hood before being incubated, to prevent running. For liquid cultures, 500 µl of DSC was used to inoculate 300-ml flasks containing 100 ml of ICI medium (Imperial Chemical Industries Ltd.) (25) containing 6 mM NH_4_NO_3_ (low-nitrogen medium) or 60 mM NH_4_NO_3_ (high-nitrogen medium). For conditioned-medium experiments, 30 ml of cell-free Ralstonia medium (either GMI1000 or Δ*rmyA* strain) was added to each flask of 100 ml ICI medium, and 30 ml of sterile CPG medium was used for control experiments. For liquid cultures of F. fujikuroi with purified ralsolamycin, purified ralsolamycin was added at a final concentration of 10 µg/ml (half the average final concentration found in the supernatant) in a 12-ml total volume. Details of DNA and RNA extraction protocols are provided in Text S1 in the supplemental material.

10.1128/mBio.00820-18.1 TEXT S1  Supplemental materials and methods. Download TEXT S1, DOCX file, 0.02 MB. Copyright © 2018 Spraker et al. 2018 Spraker et al. This content is distributed under the terms of the Creative Commons Attribution 4.0 International license.

### Imaging mass spectrometry experiments.

Bacteria and fungi were cultured as described above for solid-plate assays. The region of interest was excised from the agar and transferred to the same MALDI MSP 96 anchor plate (Bruker Daltonics, Billerica, MA). A photograph was taken, and the aerial hyphae of F. fujikuroi were removed gently with a cotton swab dampened with acetonitrile (ACN) (46). Following the removal of the aerial hyphae, another photograph was taken and a 50:50 mixture of recrystallized α-cyano-4-hydroxycinnamic acid (CHCA) and 2,5-dihydroxybenzoic acid (DHB) (Sigma-Aldrich) was applied using a 53-µm stainless steel sieve (Hogentogler & Co., Inc., Columbia, MD). The plate was then transferred to an oven and desiccated at 37°C overnight. Following adherence to the MALDI plate, another photographic image was taken and the sample was subjected to MALDI-TOF mass spectrometry (Autoflex; Bruker Daltonics, Billerica, MA) for IMS acquisition. Data were acquired in positive reflectron mode, with a 500-µm laser interval in the XY and a mass range of 200 to 2,500 Da. The resulting data were analyzed using Flex imaging software, v. 4.0.

### Metabolite extraction and quantification.

Liquid cultures were grown as described above, collected at 72 h, and extracted with 200 ml of ethyl acetate acidified with 1 ml of 25% HCl. From each sample, 15 ml of ethyl acetate layer was transferred to a scintillation vial and dried down *in vacuo*. Liquid cultures with purified ralsolamycin were collected at 72 h and extracted with 8 ml ethyl acetate acidified with 0.005% HCl, 5 ml of which was dried down *in vacuo*. Dried sample residues were reconstituted in ACN-water-formic acid (99:99:2, vol/vol/vol) and separated on a Zorbax Eclipse XDB-C_18_ column (Agilent; 4.6 mm by 150 mm with a 5-µm particle size) using a binary gradient of 1% formic acid as solvent A and ACN-1% formic acid as solvent B delivered by a Flexar binary liquid chromatography (LC) pump (PerkinElmer) coupled to a Flexar LC autosampler (PerkinElmer) and a Flexar Photodiode Array (PDA) Plus detector (PerkinElmer). The high-performance LC (HPLC) program started with an isocratic step at 80% A for 2 min followed by a linear gradient to 0% A over 15 min. After each run, the column was washed for 3 min using 100% B and was equilibrated for 3 min using 80% A. The flow rate was set to 1.5 ml min^−1^. Bikaverin was detected at 510 nm. Identification and relative quantification of metabolites were performed using Chromera Manager (PerkinElmer). Extraction and enrichment of ralsolamycin are described and nuclear magnetic resonance (NMR) experimental details are provided in Text S1 in the supplemental material.

For our UHPLC-MS-guided metabolomics experiments, conditioned medium and extraction protocols were used as described above. High-resolution UHPLC-MS was performed on a Thermo Scientific-Vanquish UHPLC system connected to a Thermo Scientific Q Exactive Orbitrap operated in electrospray negative ionization mode (ESI). A Zorbax Eclipse XDB-C_18_ (2.1 by 150 mm, 1.8-µm particle diameter) column was used for all samples with a flow rate of 0.2 ml/min. The solvent system was water with 0.5% formic acid (solvent A) and acetonitrile with 0.5% formic acid (solvent B) with the following gradient: 20% to 100% B from 0 to 15 min, 100% B from 15 to 20 min, 100% to 20% B from 20 to 21 min, and 20% B from 21 to 25 min. Nitrogen was used as the sheath gas. Data acquisition and processing for the UHPLC-MS were controlled by Thermo Scientific Xcalibur software. Further details of data processing and analyses are provided in Text S1 in the supplemental material.

### Microscopy.

Cocultures were examined using an LMS 225R dissecting scope (Olympus) and a CX31 compound scope (Olympus). Images were collected on a mounted Canon EOS Rebel T3 and processed in Adobe Photoshop CC 2015 (Adobe Inc.). For assessment of endofungal colonization by R. solanacearum, green fluorescent protein (GFP)-labeled strains of GMI1000 were cocultured with F. fujikuroi in 3 ml of CPG medium for 1 week at 28°C at 180 rpm. Resultant cultures were placed on Miracloth and washed with 15 ml double-distilled H_2_O to remove excess bacterial cells. Hyphae were collected and stained with calcofluor white at a final concentration of 1 mg/ml for 1 min prior to microscopy. Stained samples were wet mounted and imaged on a Zeiss Elyra PS.1 LSM 780 confocal laser scanning microscope (Carl Zeiss, Oberkochen Germany) equipped with a 40× 1.10C-Apochromat lens. To verify endohyphal localization of bacteria, 0.5-µm Z-sections were taken across multiple planes of view. An argon ion laser was used to excite GFP-labeled cells as well as the calcofluor white-labeled fungal cell walls. All confocal images were processed in the open-source Fiji software package, and all overlays of multiple fluorescence channels are of single confocal Z-planes.

For analyses of bacterial invasion, regions of interest (ROIs) were drawn around all calcofluor white-labeled chlamydospores. A custom macro was used to adjust thresholds to 3,500 in the GFP channel to remove background fluorescence in all images and then quantify total bacterial (GFP) fluorescence area in each ROI (https://github.com/spraker/Chlamydospore_colonization-counts). These data were copied to GraphPad Prism and analyzed for significance using a *t* test. Chlamydospores containing a contiguous fluorescence area of ≥1.5 or <1.5 µm^2^ were binned as colonized or uncolonized, respectively, for chi-square analyses.

### Antibacterial assays.

For assessing antibacterial activity of conditioned medium, 200 µl of cell-free supernatants from low-nitrogen F. fujikuroi (both wild-type and Δ*bik1*) cultures was inoculated with GMI1000 cells at a final optical density (OD) of 0.01, transferred into a 96-well plate, and grown for 48 h at 28°C. To assess bacterial survival, cells were dilution plated on CPG plus TZC and quantified microscopically. These experiments were done in triplicate, and the data were analyzed using Student’s *t* test to determine significance. To assess antibacterial activity of purified bikaverin and beauvericin (Adipogen), similar protocols were used except that bacteria were grown in Boucher’s minimal medium (47). Stock solutions of bikaverin (0.5 mg/ml) and beauvericin (5 mg/ml) were made in dimethyl sulfoxide (DMSO), and appropriate volumes were added to each well to achieve the final concentrations. Equal volumes of DMSO were added to control wells to ensure that activity was attributable to bikaverin and/or beauvericin and not the carrier solvent. For the checkerboard assay using a microdilution approach in a 96-well plate, minimum concentrations of the compounds that showed zero growth based on visible scoring and OD_600_ values after 48 h were chosen as the MICs. The sum of the fractional inhibitory concentrations (FICs) was calculated as follows for assessing the interaction: ∑FIC = FIC_A_ + FIC_B_, where FIC_A_ = (MIC of compound A in combination with B/MIC of A alone), where “MIC of A alone” is the MIC of compound A when used as the sole agent. The “MIC of compound A in combination” is the MIC of compound A when used in combination with agent B. The following values were used as cutoffs based on EUCAST recommendations: 0.5 for synergism, 0.5 to 1 for additivity, 1 to 4 for indifference, and 4 for antagonism.

### Statistical analysis.

All experiments for bikaverin quantification were carried out in biological triplicates and technical duplicates unless otherwise stated in the figure legends. Statistical analysis was performed by using GraphPad Prism software using Student’s *t* test or analysis of variance (ANOVA) followed by Tukey-Kramer analysis to show significant differences. Untargeted metabolomics analyses were performed from four replicates within the XCMS and metaXCMS software packages implemented in the R language (https://github.com/spraker/Spraker_XCMS_analysis_Fusarium). For analyses of known F. fujikuroi metabolites, the MAVEN software package (48) was used to align peaks, and the area top measurement (average intensity of the top three points of a peak) was used for statistical analyses between treatments. ANOVAs followed by Tukey *post hoc* analyses were performed using GraphPad Prism software. Confocal microscopy images were processed in Fiji, and *t* tests of bacterial fluorescence in chlamydospores were performed using GraphPad Prism.

10.1128/mBio.00820-18.9 TABLE S4  Primers used in this study. Download TABLE S4, PDF file, 0.04 MB. Copyright © 2018 Spraker et al. 2018 Spraker et al. This content is distributed under the terms of the Creative Commons Attribution 4.0 International license.
