# Supplementary figures and images for: Conserved Responses in a War of Small Molecules between a Plant-Pathogenic Bacterium and Fungi
Source: mBio. 2018 May 22;9(3):e00820-18. doi: 10.1128/mBio.00820-18 (PMC5964348; doi:10.1128/mBio.00820-18)

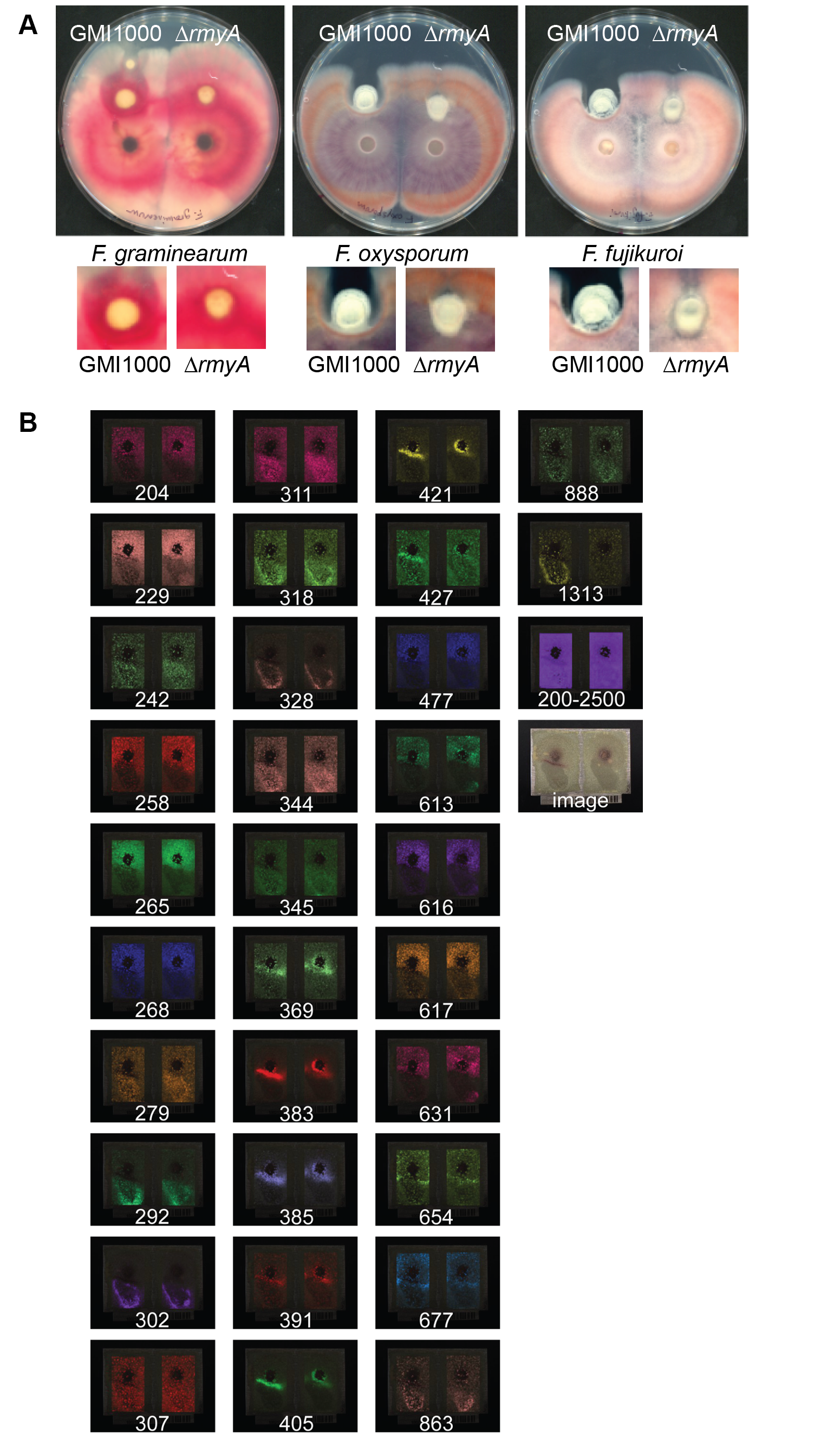

Supplement: FIG S1 [file mbo001183899sf1.tif]

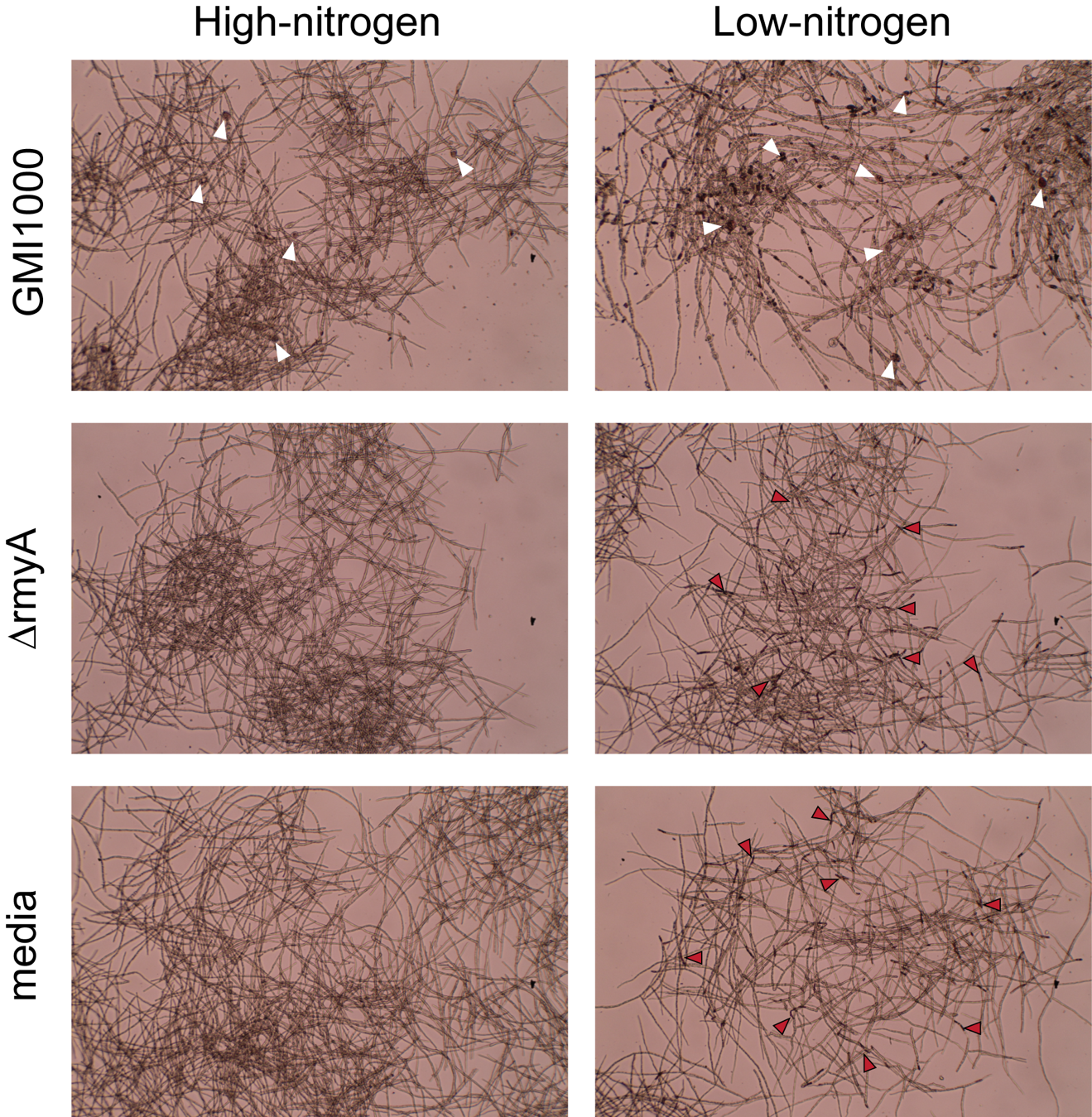

Supplement: FIG S2 [file mbo001183899sf2.tif]

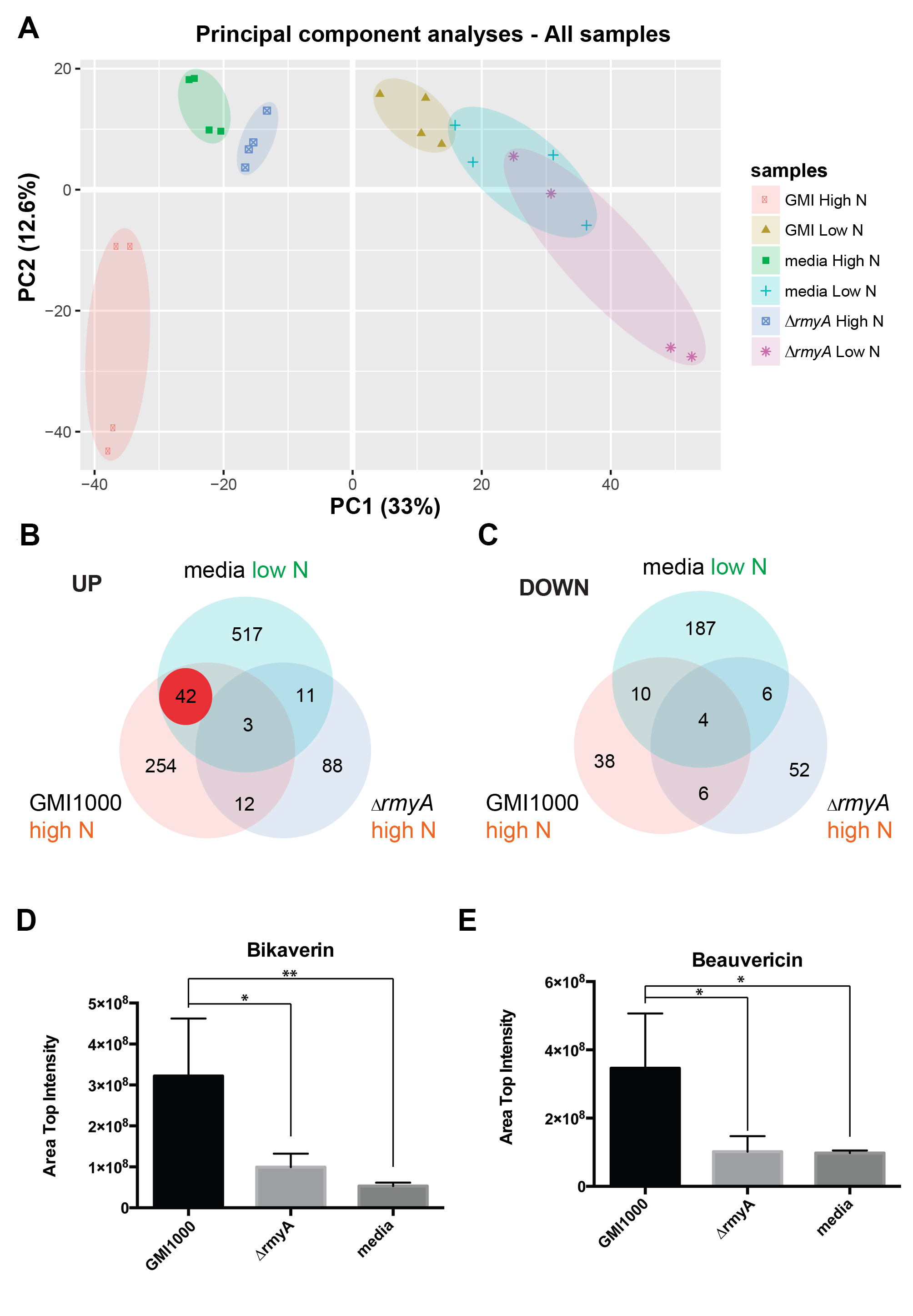

Supplement: FIG S3 [file mbo001183899sf3.tif]

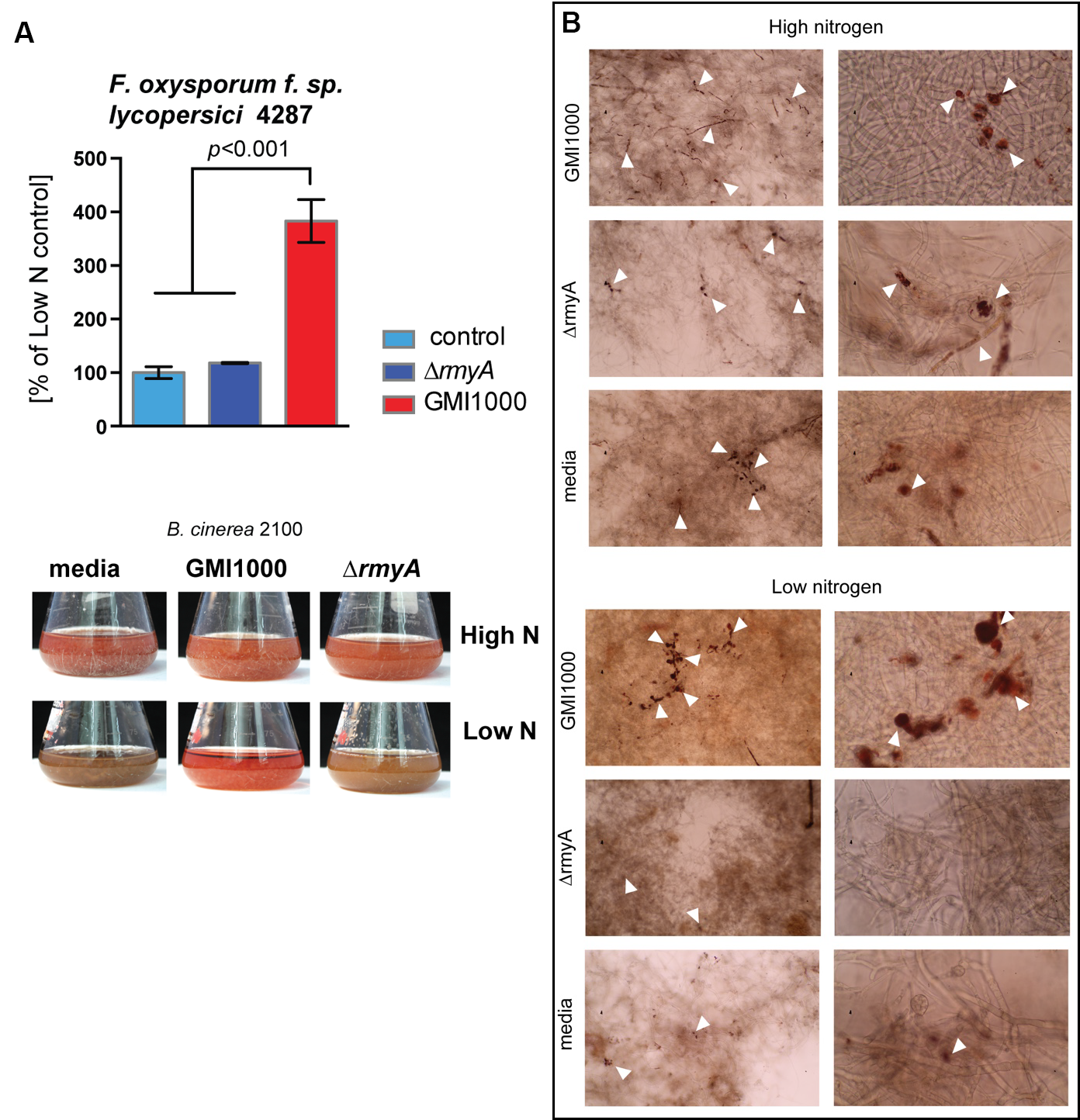

Supplement: FIG S4 [file mbo001183899sf4.tif]
