# Supplementary material for: Conserved Responses in a War of Small Molecules between a Plant-Pathogenic Bacterium and Fungi
Source: mBio. 2018 May 22;9(3):e00820-18. doi: 10.1128/mBio.00820-18 (PMC5964348; doi:10.1128/mBio.00820-18)
Supplement: DATA SET S1 [file mbo001183899sd1.pdf]

Figure A1. NMR spectra of ralsolamycin. Each panel is labeled in the top left corner with experimental detail

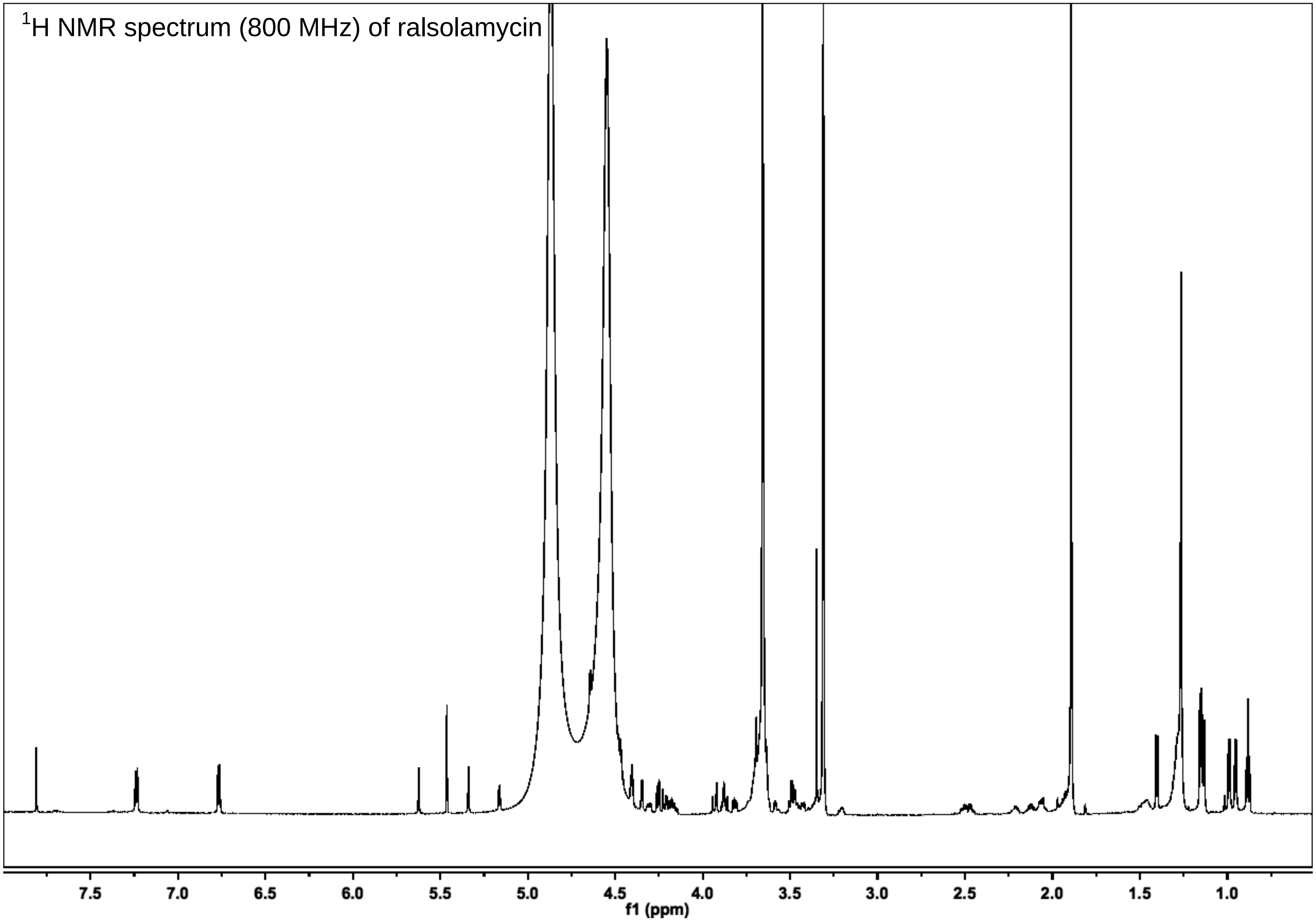

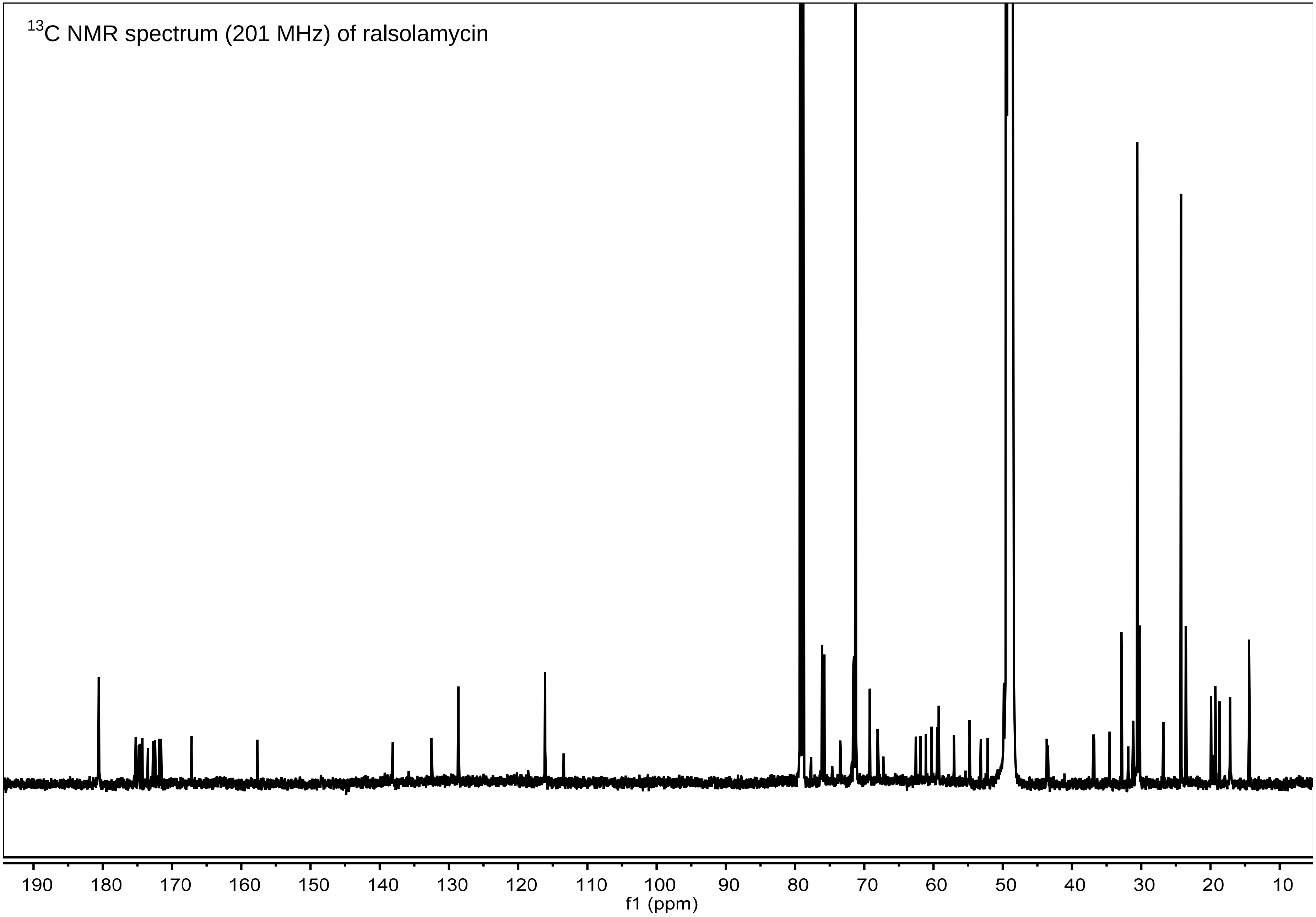

^{13}C NMR spectrum (201 MHz) of ralsolamycin
190
180
170
160
150
140
130
120
110
100
90
80
70
60
50
40
30
20
10
f1 (ppm)

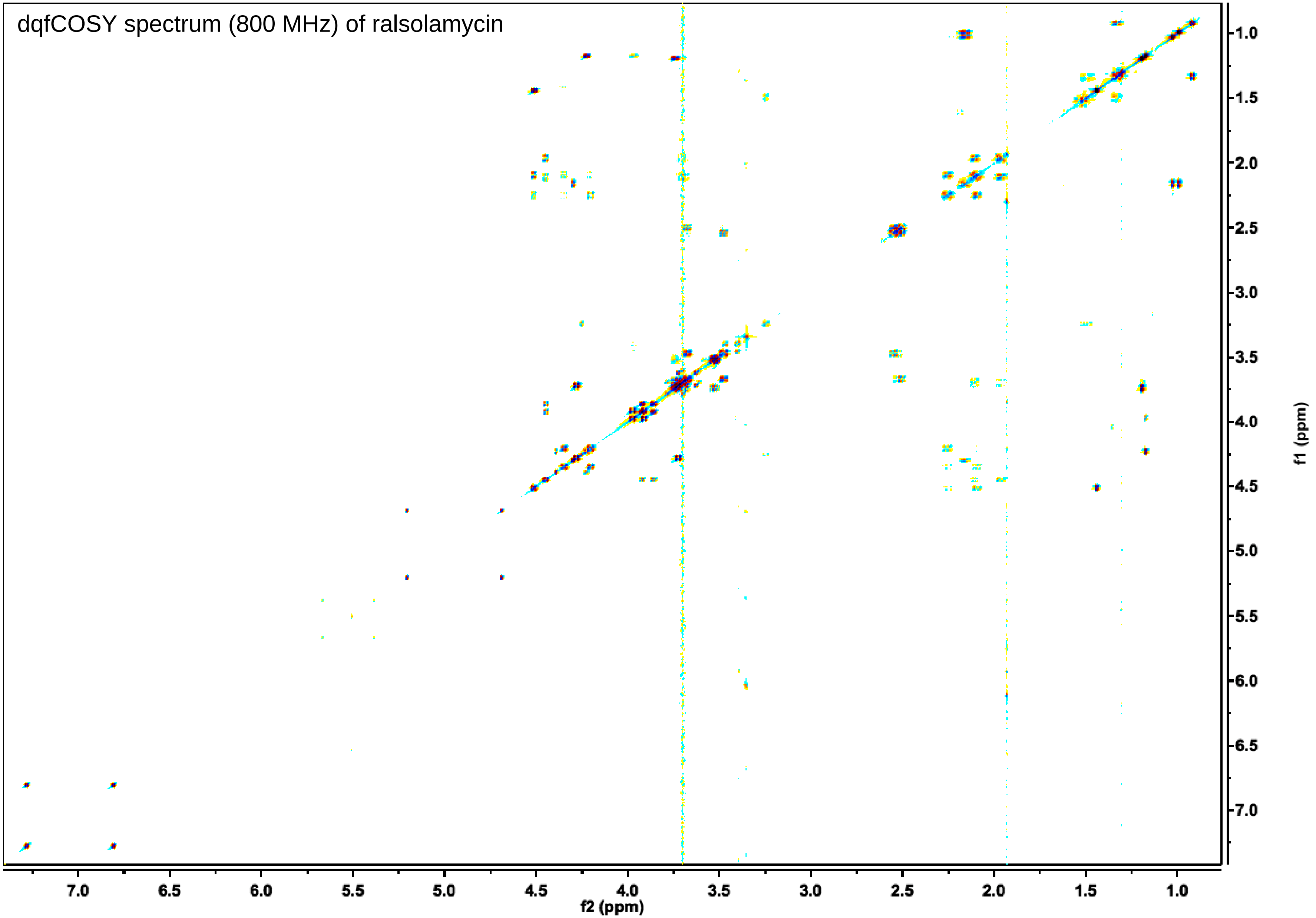
dqfCOSY spectrum (800 MHz) of ralsolamycin
f2 (ppm)
f1 (ppm)
7.0
6.5
6.0
5.5
5.0
4.5
4.0
3.5
3.0
2.5
2.0
1.5
1.0

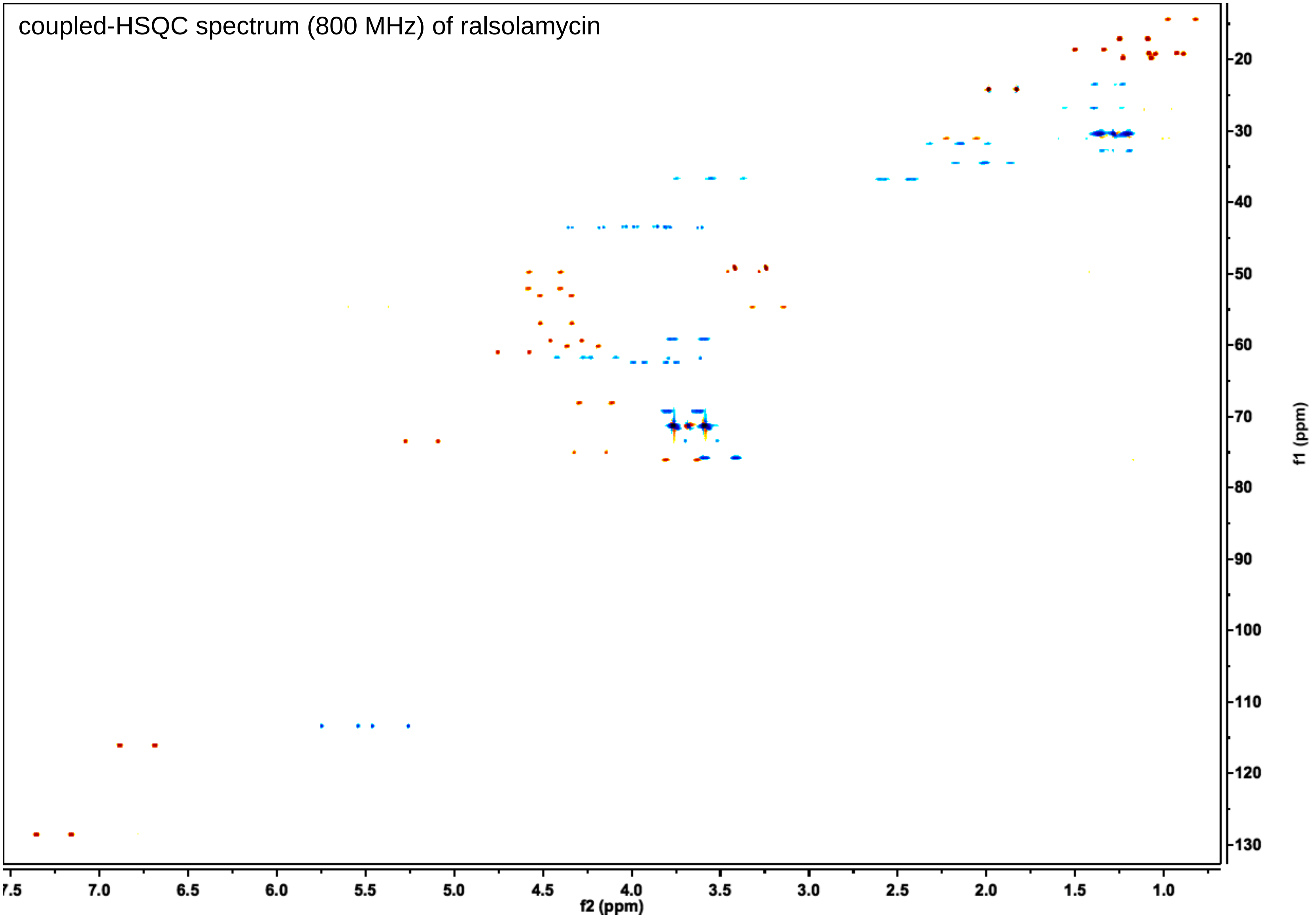
coupled-HSQC spectrum (800 MHz) of ralsolamycin
f2 (ppm)
f1 (ppm)
7.5
7.0
6.5
6.0
5.5
5.0
4.5
4.0
3.5
3.0
2.5
2.0
1.5
1.0
20
30
40
50
60
70
80
90
100
110
120
130

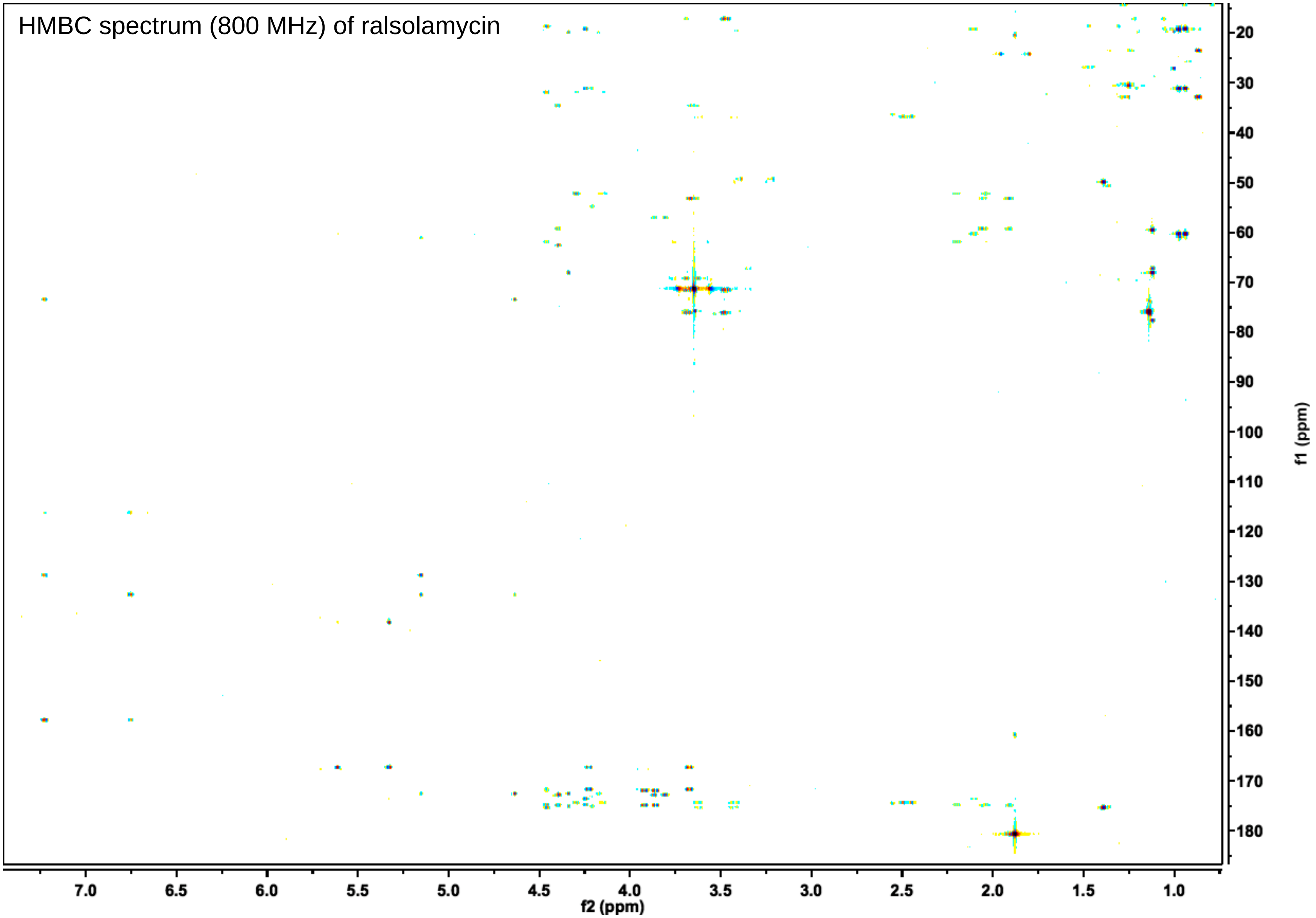

HMBC spectrum (800 MHz) of ralsolamycin
7.0
6.5
6.0
5.5
5.0
4.5
4.0
3.5
3.0
2.5
2.0
1.5
1.0
f2 (ppm)
20
30
40
50
60
70
80
90
100
110
120
130
140
150
160
170
180
f1 (ppm)

Table A1. ^{1}H (800 MHz) and ^{13}C (201 MHz) NMR spectroscopic data for ralsolamycin in CD_3OD, D_2O, and $CDCl_3$ (70:30:1, respectively)

Chemical shifts were referenced to $\delta(C\underline{H}D_2OD) = 3.31$ ppm and $\delta(^{13}\underline{C}HD_2OD_3) = 49.00$. ^{13}C chemical shifts were determined via HMBC, HSQC and direct observation ^{13}C spectra. ^{1}H, ^{1}H-J-coupling constants were determined from the acquired ^{1}H or dqfCOSY spectra. HMBC correlations are from the proton(s) stated to the indicated ^{13}C atom.

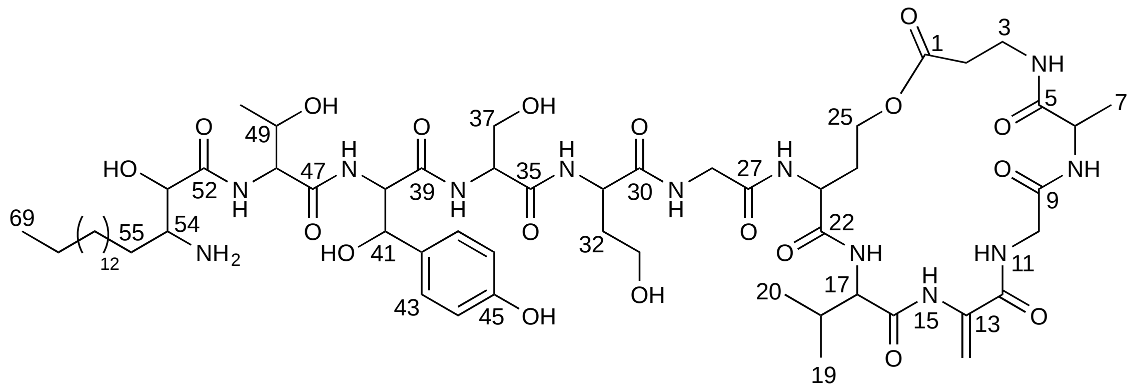

No.	δ_c	Proton	$\delta H(J_{HH}[Hz])$	HMBC
1	174.28			
2	36.90	2-H_a	2.47 ($J_{2a,2b} = 15.0$) ($J_{2a,3a} = 2.5$) ($J_{2a,3b} = 8.0$)	1,3
		2-H_b	2.52 ($J_{2b,2a} = 15.0$) ($J_{2b,3a} = 8.5$) ($J_{2b,3b} = 2.5$)	1,3
3	36.78	3-H_a	3.45 ($J_{3a,3b} = 14.6$) ($J_{3a,2a} = 2.5$) ($J_{3a,2b} = 8.5$)	2,5
		3-H_b	3.65 ($J_{3b,3a} = 14.6$) ($J_{3b,2a} = 8.0$) ($J_{3b,2b} = 2.5$)	2,5
4		4-NH		

5	175.23			
6	49.90	6-H	4.48 ($J_{6,7}$ = 7.2)	5,7,9
7	18.70	7-H_3	1.40 ($J_{7,6}$ = 7.2)	5,6
8		8-NH		
9	171.57			
10	43.63	10-H_a	3.69 ($J_{10a,10b}$ = 17.4)	9,12
		10-H_b	4.25 ($J_{10b,10a}$ = 17.4)	9,12
11		11-NH		
12	167.18			
13	137.22			
14	113.40	14-H_a	5.35 ($J_{14a,14b}$ = 1.0)	12
		14-H_b	5.64 ($J_{14b,14a}$ = 1.0)	12
15		15-NH		
16	173.47			
17	60.27	17-H	4.27 ($J_{17,18}$ = 9.0)	16,18,19,20,21,22
18	31.17	18-H	2.14 ($J_{18,17}$ = 9.0) ($J_{18,19}$ = 6.8) ($J_{18,20}$ = 6.8)	17,19,20
19	19.29	19-H_3	0.96 ($J_{19,18}$ = 6.8)	17,18
20	19.22	20-H_3	1.00 ($J_{20,18}$ = 6.8)	17,18
21		21-NH		
22	174.68			
23	52.19	23-H	4.49 ($J_{23,24a}$ = 9.6) ($J_{23,24b}$ = 5.2)	22,25,27
24	31.89	24-H_a	2.08 ($J_{24a,23}$ = 9.6)	22

		24-H_b	2.23	22
25	61.89	25-H_a	4.18 ($J_{25a,25b}$ = 12.0)	1,
		25-H_b	4.32 ($J_{25b,25a}$ = 12.0)	1,
26		26-NH		
27	171.86			
28	43.51	28-H_a	3.89 ($J_{28a,28b}$ = 16.5)	27,30
		28-H_b	3.95 ($J_{28a,28b}$ = 16.5)	27,30
29		29-NH		
30	174.72			
31	53.19	31-H	4.42 ($J_{31,32a}$ = 8.9) ($J_{31,32b}$ = 4.8)	30
32	34.58	32-H_a	1.94 ($J_{32a,31}$ = 8.9)	30,33
		32-H_b	2.09 ($J_{32b,31}$ = 4.8)	30,33
33	59.20	33-H_2	3.68	
34		34-NH		
35	172.72			
36	57.02	36-H	4.42 ($J_{36,37a}$ = 5.4) ($J_{36,37b}$ = 5.4)	35,39
37	62.59	37-H_a	3.82 ($J_{37a,37b}$ = 11.3) ($J_{37a,36}$ = 5.4)	35
		37-H_b	3.90 ($J_{37b,37a}$ = 11.3) ($J_{37b,36}$ = 5.4)	35
38		38-NH		
39	172.48			
40	61.09	40-H	4.66 ($J_{40,41}$ = 4.4)	39,42,47
41	73.47	41-H	5.18 ($J_{41,40}$ = 4.4)	39,42,43

42	134.57			
43	128.66	43-H_2	7.26 ($J_{43,44}$ = 8.5)	41,43,45
44	116.11	44-H_2	6.78 ($J_{44,43}$ = 8.5)	42,44
45	157.69			
46		46-NH		
47	172.46			
48	59.51	48-H	4.36 ($J_{48,49}$ = 3.9)	47,49,50,52
49	68.07	49-H	4.20 ($J_{49,48}$ = 3.9) ($J_{49,50}$ = 6.4)	52
50	19.88	50-H_3	1.15 ($J_{50,49}$ = 6.4)	
51		51-NH		
52	174.96			
53	74.98	53-H	4.23 ($J_{53,54}$ = 4.7)	52,54
54	54.81	54-H	3.22 ($J_{54,53}$ = 4.7)	
55	32.17	55-H_2	1.46	
56-66*	30.50	56-67-H_{22}	1.27	
67	32.80	67-H_2	1.27	
68	23.53	68-H_2	1.31 ($J_{68,69}$ = 7.2)	
69	13.43	69-H_3	0.88 ($J_{69,68}$ = 7.2)	67,68
