# Supplementary material for: Conserved Responses in a War of Small Molecules between a Plant-Pathogenic Bacterium and Fungi
Source: mBio. 2018 May 22;9(3):e00820-18. doi: 10.1128/mBio.00820-18 (PMC5964348; doi:10.1128/mBio.00820-18)
Supplement: TABLE S1 [file mbo001183899st1.pdf]

	Bac+	Bac-	Totals
WT	3	151	154
Δbik1	20	281	301
Totals	23	432	455

K (degrees of freedom) = 1
X^2 stat = 4.682
p = 0.0305
