# Supplementary material for: Conserved Responses in a War of Small Molecules between a Plant-Pathogenic Bacterium and Fungi
Source: mBio. 2018 May 22;9(3):e00820-18. doi: 10.1128/mBio.00820-18 (PMC5964348; doi:10.1128/mBio.00820-18)
Supplement: TABLE S2 [file mbo001183899st2.pdf]

Compound	Molecular Weight	Formula	ANOVA p
Moniliformin	98.06	$C_4H_2O_3$	na
Beauvericin	783.96	$C_{45}H_{57}N_3O_9$	0.0079
Fusarubin	306.27	$C_{15}H_{14}O_7$	na
Fumonisin B1	721.84	$C_{34}H_{59}NO_{15}$	0.0687
Fumonisin B2	705.83	$C_{34}H_{59}NO_{14}$	na
Fusaric acid	179.22	$C_{10}H_{13}NO_2$	na
Bikaverin	382.32	$C_{20}H_{14}O_8$	0.003
Fusarin C	431.49	$C_{23}H_{29}NO_7$	0.1043
GA3	346.38	$C_{19}H_{22}O_6$	na
GA4	332.4	$C_{19}H_{24}O_5$	0.1587
GA7	330.38	$C_{19}H_{22}O_5$	0.0914
Apicidin	622.8	$C_{34}H_{48}N_5O_6$	na
Apicidin-like	645.3	$C_{35}H_{42}O_7N_5$	na
Fujikurin A	224.25	$C_{12}H_{16}O_4$	na
Fujikurin B	242.27	$C_{12}H_{18}O_5$	na
Fujikurin C/D	226.27	$C_{12}H_{18}O_4$	0.3155
Caryophyllene	204.35	$C_{15}H_{24}$	0.3268
Neurosporaxanthin	498.74	$C_{35}H_{46}O_2$	na
α-acorenol	222.37	$C_{15}H_{26}O$	0.3184
