# Supplementary material for: Conserved Responses in a War of Small Molecules between a Plant-Pathogenic Bacterium and Fungi
Source: mBio. 2018 May 22;9(3):e00820-18. doi: 10.1128/mBio.00820-18 (PMC5964348; doi:10.1128/mBio.00820-18)
Supplement: TABLE S3 [file mbo001183899st3.pdf]

Isolate tested	Test Agent	Minimum Inhibitory Concentration (µg/ml)				∑FIC$_{mean}$[c]	FIC interpretation
		Alone[a]	In combination[b]				
***Ralstonia solanacearum* GMI1000**	Bikaverin	60	**7.5**	**15**	**30**	0.708	**Additive**
	Beauvericin	240	**120**	**120**	**60**		
