# Supplementary material for: Conserved Responses in a War of Small Molecules between a Plant-Pathogenic Bacterium and Fungi
Source: mBio. 2018 May 22;9(3):e00820-18. doi: 10.1128/mBio.00820-18 (PMC5964348; doi:10.1128/mBio.00820-18)
Supplement: TEXT S1 [file mbo001183899s1.docx]

**Title:**

**Conserved responses in a war of small molecules between a plant pathogenic bacterium and fungi**

**Running title:**

**Conserved microbial small molecule warfare**

Joseph E. Spraker^a,†,*^, Philipp Wiemann^b,†,*^, Joshua A. Baccile^c,*^, Nandhitha Venkatesh^a^, Julia Schumacher^d^, Frank C. Schroeder^e^, Laura M. Sanchez^f^, Nancy P. Keller^b,f,#^

^a^Department of Plant Pathology, University of Wisconsin - Madison, Madison, WI, United States

^b^Department of Medical Microbiology and Immunology, University of Wisconsin - Madison, Madison, WI, United States

^c^Boyce Thompson Institute and Department of Chemistry and Chemical Biology, Cornell University, Ithaca, NY, United States

^d^Institute for Biology and Biotechnology of Plants, Westfälische Wilhelms-Universität Münster, Münster, Germany

^e^Department of Medicinal Chemistry and Pharmacognosy, College of Pharmacy, University of Illinois at Chicago, Chicago, IL, United States

^f^Department of Bacteriology, University of Wisconsin - Madison, Madison, WI, United States

^†^Contributed equally to the preparation of this manuscript

^#^Corresponding author ([npkeller@wisc.edu)](mailto:npkeller@wisc.edu))

**^*^Current address:**

JES – School of Plant Sciences, University of Arizona, Tucson, AZ, United States.

JAB – Division of Chemistry and Chemical Engineering, California Institute of Technology, Pasadena, CA, United States.

PW – Hexagon Bio, Menlo Park, CA, United States

**SUPPLEMENTARY MATERIALS AND METHODS**

**DNA isolation, RNA extraction and expression analysis**

For DNA isolation, *F. fujikuroi* mycelia was collected from 48 h culture in Darken Media and immediately freeze dried. DNA isolation was performed from dried mycelia as described previously (Green and Sambrook, 2012). For RNA extraction, mycelia were harvested by filtration through Miracloth (Calbiochem) at 24, 48, and 72 h, respectively, and immediately freeze dried. Dried mycelia were pulverized and total RNA was extracted with TRIzol reagent (Invitrogen) following the manufacturer's protocol. Total RNA was quantified using a NanoDrop spectrophotometer (Thermo Scientific). To assess expression, cDNA was generated from 1 µg of DNAse treated total RNA using the iScript cDNA synthesis kit (BioRad) according to the manufacturer's protocol. Primers for semi-quantitative PCR were designed to areas internal to the gene of interest and span introns when present and are listed in **Table S4** (“gene”-F/ “gene”-R).

**UHPLC-MS Data Processing and Analysis**

Files were converted to .mzXML using MassMatrix MS Data File Conversion grouped by condition, and run in the XCMS package in R (Colin A Smith *et al.*, 2006). This method allows for the identification of metabolites across samples without the use of internal standards. Using non-linear retention time correction for each sample, extracted ion intensity profiles can be compared between samples, showing which metabolites are differentially produced (either over- or under-represented) The R-script used in these analyses is available upon request.

To evaluate differentially produced compounds between the GMI1000 and ∆*rmyA* conditioned media, pairwise analyses were carried out in XCMS (R script available upon request). Briefly, each bacterial treatment (either GMI100 or ∆*rmyA*) was analyzed relative to the appropriate media control matching the nitrogen condition. Additionally, high- and low-N media conditions were compared to differentiate signals resulting from abiotic (nitrogen availability) vs biotic (bacterial metabolites), helping to reduce the complexity of the dataset. Subsequently, these pairwise analyses were analyzed in the program metaXCMS (Patti *et al.*, 2012) to identify differentially produced metabolites amongst all of the datasets. In metaXCMS metabolites were first filtered using a fold-change of ≥5 and a *p*-value cutoff of 0.05. To assign common features for generating Venn diagrams we used an *m/z* tolerance of 0.001 and a retention time (RT) tolerance of 30 seconds. Common features were extracted from metaXCMS and analyzed manually to identify putative isotopes and adducts.

**Nuclear magnetic resonance Spectroscopy**

Nuclear magnetic resonance (NMR) spectroscopic instrumentation: Bruker Avance^III^ HD (800 MHz ^1^H reference frequency, 201 MHz for ^13^C) equipped with a cryo probe. Non-gradient phase-cycled dqfCOSY spectra were acquired using the following parameters: 0.6 s acquisition time, 675 complex increments, 8 scans per increment. Non-gradient HSQC and HMBC spectra were acquired with these parameters: 0.25 s acquisition time, 500 increments, 24 and 56 scans per increment, respectively. ^1^H, ^13^C-HMBC spectra were optimized for J_H,C_ = 6 Hz. NMR spectra were processed and baseline corrected using MestreLabs MNOVA software packages.

**Chromatographic enrichment of ralsolamycin**

Bulk extracts of ralsolamycin were isolated from liquid culture by growing GMI1000 for four days in two separate 1 L volumes of liquid CPG with 100 g Amberlite XAD-16 resin (Sigma- Aldrich). The resin was separated from the culture broth via vacuum filtration and rinsed with 500 mL double-distilled H_2_O to remove excess salts and cellular debris. The adsorbed compounds were eluted in 1:1 methanol/dichloromethane (v/v) and organic fractions were evaporated to dryness and stored in 20 mL vials. Fractions containing ralsolamycin were further purified via semi-preparative HPLC using an Agilent XDB C-18 (25 cm x 10 mm, 5 μm particle diameter) acetonitrile (organic phase) and 0.1 % acetic acid in water (aqueous phase) as solvents at a flow rate of 3.6 mL/min. A solvent gradient scheme was used, starting at 5% organic for 3 min, followed by a linear increase to 100% organic over 27 min, holding at 100% organic for 5 min, then decreasing back to 5% organic for 0.1 min and holding at 5% organic for the final 4.9 min, a total of 40 min.
