# Supplementary material for: Conserved Responses in a War of Small Molecules between a Plant-Pathogenic Bacterium and Fungi
Source: mBio. 2018 May 22;9(3):e00820-18. doi: 10.1128/mBio.00820-18 (PMC5964348; doi:10.1128/mBio.00820-18)
Supplement: TABLE S4 [file mbo001183899st4.pdf]

Primer name	Sequence (5’-3’)	Use
bik1-F	TCGAATCAGGAGGAGCTTGT	Semiquantitative PCR
bik1-R	GACCATAGATGGGAGCTGGA	Semiquantitative PCR
bik2-F	TTGATCAGCTTGGTCTCGTG	Semiquantitative PCR
bik2-R	GGTTCAGGCAGCTTTGAGTC	Semiquantitative PCR
bik3-F	CGCAGGTTGCTAAGGAGAAG	Semiquantitative PCR
bik3-R	AGCATTGAAAGCTGCCTTGT	Semiquantitative PCR
actA-F	CCTGCTTGGAGATCCACATT	Semiquantitative PCR
actA-R	CTTCCAGCCTTCTGTCCTTG	Semiquantitative PCR
